# Development of human targeted extracellular vesicles loaded with shRNA minicircles to prevent parkinsonian pathology

**DOI:** 10.1186/s40035-025-00484-7

**Published:** 2025-05-26

**Authors:** Maria Izco, Carlos Sola, Martin Schleef, Marco Schmeer, María de Toro, Guglielmo Verona, Estefania Carlos, Alejandro Reinares-Sebastian, Sandra Colina, Maria Eugenia Marzo-Sola, Josune Garcia-Sanmartin, Joaquín Fernández-Irigoyen, Enrique Santamaría, Rodolfo Mugica-Vidal, Javier Blesa, Lydia Alvarez-Erviti

**Affiliations:** 1 Laboratory of Molecular Neurobiology, Center for Biomedical Research of La Rioja (CIBIR), 26006 Logroño, Spain; 2 Transfusion Center and Blood Bank of La Rioja, 26006 Logroño, Spain; 3 PlasmidFactory GmbH, 33607 Bielefeld, Germany; 4 Genomics and Bioinformatics Core Facility, Center for Biomedical Research of La Rioja (CIBIR), 26006 Logroño, Spain; 5 Centre for Amyloidosis, UCL Medical School, Rowland Hill Street, London, NW3 2PF UK; 6 HM CINAC (Centro Integral de Neurociencias Abarca Campal), Hospital Universitario HM Puerta del Sur, HM Hospitales, Madrid, Spain; 7 Instituto de Investigación Sanitaria HM Hospitales, Madrid, Spain; 8 Servicio de Neurología, Hospital San Pedro, Piqueras 98, 26006 Logroño, Spain; 9 Angiogenesis Group, Oncology Area, Center for Biomedical Research of La Rioja (CIBIR), 26006 Logroño, Spain; 10 Clinical Neuroproteomics Unit, Proteomics Platform, Navarrabiomed, Hospitalario, Universitario de Navarra (HUN), 31008 Pamplona, Spain; 11 Department of Mechanical Engineering, University of La Rioja, 26004 Logroño, Spain

**Keywords:** Parkinson’s disease, Gene therapy, α-Synuclein, Human targeted extracellular vesicles, ShRNA minicircles

## Abstract

**Background:**

Neurological disorders are the second leading cause of death and the leading cause of disability in the world**.** Thus, the development of novel disease-modifying strategies is clearly warranted. We have previously developed a therapeutic approach using mouse targeted rabies virus glycoprotein (RVG) extracellular vesicles (EVs) to deliver minicircles (MCs) expressing shRNA (shRNA-MCs) to induce long-term α-synuclein down-regulation. Although the previous therapy successfully reduced the pathology, the clinical translation was extremely unlikely since they were mouse extracellular vesicles.

**Methods:**

To overcome this limitation, we developed a source of human RVG-EVs compatible with a personalized therapy using immature dendritic cells. Human peripheral blood monocytes were differentiated in vitro into immature dendritic cells, which were transfected to express the RVG peptide. RVG-EVs containing shRNA-MCs, loaded by electroporation, were injected intravenously in the α-synuclein performed fibril (PFF) mouse model. Level of α-synuclein, phosphorylated α-synuclein aggregates, dopaminergic neurons and motor function were evaluated 90 days after the treatment. To confirm that EVs derived from patients were suitable as a vehicle, proteomic analysis of EVs derived from control, initial and advanced Parkinson’s disease was performed.

**Results:**

The shRNA-MCs could be successfully loaded into human RVG-EVs and downregulate α-synuclein in SH-SY5Y cells. Intravenous injection of the shRNA-MC-loaded RVG-EVs induced long-term downregulation of α-synuclein mRNA expression and protein level, decreased α-synuclein aggregates, prevented dopaminergic cell death and ameliorated motor impairment in the α-synuclein PFF mouse model. Moreover, we confirmed that the EVs from PD patients are suitable as a personalized therapeutic vehicle.

**Conclusion:**

Our study confirmed the therapeutic potential of shRNA-MCs delivered by human RVG-EVs for long-term treatment of neurodegenerative diseases. These results pave the way for clinical use of this approach.

**Supplementary Information:**

The online version contains supplementary material available at 10.1186/s40035-025-00484-7.

## Introduction

Neurodegenerative diseases are the second leading cause of death and the leading cause of disability in the world. Due to the high incidence and severity of these diseases and the lack of effective treatments to slow or stop disease progression, development of new treatments is becoming one of the greatest sanitary challenges of the century.

Parkinson’s disease (PD) is the second most common neurodegenerative disease. From 1990 to 2015, the number of individuals with PD globally increased by 118% to 6.2 million [1]. By 2040, the number of people with PD is projected to exceed 12 million worldwide [2]. PD treatments mainly address the motor symptoms and dopaminergic therapies remain the gold standard for symptomatic management of PD. Moreover, as the disease progresses, the drugs become less effective and are associated with significant side effects. Thus, the development of novel disease-modifying strategies is clearly warranted. The primary cause of PD in the majority of patients is unknown. However, multiple lines of evidence support the central role of α-synuclein in the initiation and progression of PD pathogenesis [3–6]. Consequently, therapeutic strategies targeting α-synuclein are a powerful approach for the prevention and treatment of PD [7, 8]. Several approaches are under development and validation, including compounds to decrease α-synuclein [9], passive and active immunotherapies targeting α-synuclein [10] and gene therapy.

Gene therapy has become a promising therapeutic option for PD treatment, with several clinical trials currently registered in ClinicalTrials.gov involving PD, although none was designed to modulate α-synuclein expression. A key challenge for neuronal α-synuclein gene therapy is the development of a delivery vehicle that meets these criteria: (1) ability to cross the blood–brain barrier to target the brain after peripheral administration, (2) must be immunologically inert, and (3) allows repeated administration to be effective in the long-term given the chronic condition of PD. The local delivery of adeno-associated viral vectors is the most advanced approach with several clinical trials [11]; however, the approach has several limitations, including the requirement for stereotactic surgery, delivery into a limited brain area, and development of an immune response. A non-viral vector approach using liposomes for transvascular gene therapy has been assessed in animal models [12]; however, its clinical use is limited due to immune recognition, which implies a rapid clearance and a decreased efficacy when the treatment is re-administered, preventing prolonged therapy.

To overcome these limitations, we have developed a novel gene therapy approach based on targeted extracellular vesicles (EVs), by expressing the brain-targeting rabies virus glycoprotein (RVG) peptide on the exterior surface of the EVs (RVG-EV). This approach is capable of delivering small interfering RNA (siRNA) and short hairpin RNA (shRNA) minicircles (MCs) specifically into the central nervous system after intravenous (iv) administration, resulting in gene silencing [13–15]. In addition, we have demonstrated that un-targeted EVs (control EVs) or empty RVG-EVs do not induce down-regulation in the brain or any other organs analyzed [13]. Moreover, we labeled EVs with a lipophilic IRDye (DiR) and assessed the influence of the RVG ligand on the biodistribution by comparing RVG- and non-RVG-targeted dendritic cell (DC) EVs injected iv in mice [16]. The RVG-EVs display a significantly increased signal in brain compared to the non RVG-EVs group.

An important requirement for PD gene therapy targeting α-synuclein is to extend the duration of down-regulation from days to several weeks, which is possible by using plasmids expressing a shRNA. However, conventional plasmids are extremely difficult to be loaded into EVs due to their relatively large sizes. Alternatively, it is possible to use MCs to express shRNA. MCs have several advantages associated with the lack of additional bacterial sequences, including smaller size [17] and higher transgene expression for longer periods [18]. Recently, we have demonstrated the potential of α-synuclein shRNA-MCs (syn-MCs) loaded into mouse RVG-EVs to induce long-term down-regulation of α-synuclein expression in the brains of α-synuclein performed fibril (PFF) mouse model of PD [19]. In addition, syn-MCs also decrease α-synuclein aggregates, prevent continuing loss of dopaminergic neurons in the substantia nigra pars compacta (SNc) and ameliorate motor impairments in this model. Moreover, the multidose treatment with shRNA-MCs delivered by RVG-EVs does not activate immune responses or have any off-target effects, showing low immunogenicity and allowing repeated administrations [15]. These results highlight the therapeutic potential of the syn-MC RVG-EVs approach for disease-modifying treatment of PD.

The translation of the therapy into clinical practice requires the development of a source of modified, immunologically inert EVs compatible with personalized therapy. EVs produced by human dendritic cells (hDCs) differentiated from blood monocytes have already been successfully tested in clinical trials [20, 21]; therefore, the use of blood monocytes differentiated in vitro into immature DCs as a source of modified EVs could be a promising strategy.

In this study, we aimed to develop a RVG-EV technology for human use and assess the efficacy of the therapy in vitro and in vivo using the PFF mouse model. We used RVG-EVs derived from hDCs (h-RVG-EVs) as a vehicle for the delivery of shRNA-MCs and compared the human EVs with RVG-EVs derived from mouse DCs (Ms-RVG-EV).

## Materials and methods

### Study design

The overall objective of the study was to develop a source of modified EVs compatible with a personalized therapy and assess the therapeutic potential in a progressive mouse model of PD. We first optimized the production and transfection of human immature DCs differentiated from monocytes recovered from leukocyte depletion filters and characterized the production of targeted EVs. In vitro studies using SH-SY5Y cells were designed to assess whether h-RVG-EVs loaded with shRNA-MCs would be able to specifically deliver their cargo and down-regulate α-synuclein. For in vivo experiments, mice received intrastriatal injection of murine α-synuclein PFFs, and then treated with h-RVG-EVs containing shRNA-MCs to evaluate the efficacy of the therapy. Studies with blood samples from PD patients were aimed to assess the feasibility of PD-derived EVs.

All studies involving human samples were approved by the Institutional Ethical Committee (CEImLar, protocol number: P.I.374). All animal studies involving mice followed the guidelines of the National Institutes of Health Guide for the Care and Use of Laboratory Animals and were approved by the ethical committee on animal welfare at our institution (Center for Biomedical Research of La Rioja, ref LAE-03). To ensure reproducibility, all mice were randomly assigned to experimental groups by a person not involved in the study. The sample sizes are specified in the corresponding figure legend along with the details of statistical analysis and were estimated by power analysis using the G*Power software. The investigators were blinded to the experimental groups when assessing behavioral tests and performing experimental procedures including immunohistochemistry, western blot or qPCR.

## Cell culture

Human SH-SY5Y neuroblastoma cell line (American Type Culture Collection) clone constitutively expressing human wild-type α-synuclein with a C-terminal hemagglutinin (HA) tag was cultured using standard conditions [15].

## Dendritic cell culture and EV isolation

Peripheral blood mononuclear cells (PBMCs) were isolated from discarded blood donation fractions, in which 50 mL of PBS was added and PBMCs were separated using a density gradient (Ficoll-Paque PREMIUN 1.073, Cytiva Europe GmbH, München, Germany). Alternatively, PBMCs were isolated from blood samples of controls and PD patients (15 mL), using a density gradient (Ficoll-Paque PREMIUN 1.073, Cytiva). Monocytes were isolated form PBMCs and cultured (5 × 10^7^ cells per plate, 10-cm plate) in RPMI-1640 with GlutaMAX (GIBCO, Waltham, MA), 10% fetal calf serum (FCS) depleted of EVs by centrifugation at 120,000× *g* for 60 min, and penicillin/streptomycin. Two hours later, the medium was removed and replaced with fresh medium supplemented with 100 ng/mL human GM-CSF (Preprotech, Waltham, MA) and 20 ng/mL human IL-4 (Preprotech). The hDCs were characterized by microscopy and specific markers CD80, CD86 and MHC-II. Then 10^6^ hDCs were electroporated after 7 days with 50 µg of h-RVG-Lamp2b mRNA (300 V-150 mA). After 24 h, the medium was removed, and EVs were harvested by centrifugation at 12,000× *g* for 30 min to remove cell debris. The supernatant was centrifuged again at 120,000× *g* for 1 h to pellet EVs. EVs were resuspended in 0.1 mol/L ammonium acetate with a 27G needle. EV size distribution and concentration were assessed by nanoparticle tracker analysis using a NS500 instrument (Nanosight).

## Loading of RVG-EVs with shRNA-MCs

For in vitro use, 5 × 10^8^ RVG-EVs were mixed in 100 μL electroporation buffer (1.15 mmol/L potassium phosphate [pH 7.2], 25 mmol/L KCl, 21% OptiPrep) with 1 μg shRNA-MCs and electroporated (450 V, 100 mA) in a 4-mm cuvette using a Bio-Rad Gene Pulser Xcell Electroporator. Samples were treated with 1 U of DNase (Promega, Madison, WI) for 30 min at 37 °C and purified by ultracentrifugation at 120,000× *g* for 1 h. EVs were resuspended in the RPMI medium. For in vivo injection, 10^10^ EVs and 150 μg shRNA-MCs were mixed in 10 mL electroporation buffer (1.15 mmol/L potassium phosphate [pH 7.2], 25 mmol/L KCl, 21% OptiPrep) and electroporated (450 V, 100 mA) in a 4-mm cuvette using a Bio-Rad Gene Pulser Xcell Electroporator. Samples were treated with 150 U DNase (Promega) for 30 min at 37 °C and purified by ultracentrifugation at 120,000× *g* for 1 h. EVs were resuspended in 5% glucose.

## Transmission electron microscope

EVs were resuspended in water. A 10 μL drop of each sample was placed on Parafilm on a petri dish. Freshly glow-discharged (30 s, 15 mA using Leica EM ACE200 equipment) carbon-coated 200-mesh copper grids (Agar Scientific Supplies, Rotherham, UK) were incubated for 5 min on the sample drops. Grids were then stained with 8 µL of 2% uranyl acetate in water by adding small drops, held tight with tweezers, and then removed until the last drop was left for 1 min. Finally, the excess was removed by contacting the grid edge with filter. The grids were then stored in an EM grid box in a desiccator for future observation by a Tecnai T20 (Thermo Fisher Scientific, Waltham, MA) microscope at 200 kV.

## Animals

Adult male C57BL6/C3H F1 mice of 8–9 weeks old were purchased from Charles River Laboratories (Wilmington, MA). Mice were housed under environmentally controlled standard conditions with a 12-h light/dark cycle and provided with ad libitum food and water diet. Every effort was made to minimize the number of animals used and their suffering.

Mice received an injection of sonicated murine α-synuclein PFFs into the dorsal striatum. On days 2 and 45 after PFF injection, they received iv (tail vein) injection of human RVG-EVs containing syn-MCs or GFP-MCs or iv injection of vehicle (5% glucose). Another group of α-synuclein PFF-injected animals received iv injections of Ms-RVG-EVs containing syn-MCs. Control animals were injected with an equal volume of sterile PBS in the striatum and received two iv injections of vehicle. Mice were anesthetized with isoflurane, perfused with PBS and 4% paraformaldehyde or decapitated 90 days post injection (dpi).

## Preparation of mouse wild-type α-synuclein PFFs

Mouse α-synuclein purified monomers were assembled into filaments by incubation at 37 °C at 5 mg/mL in sterile PBS (pH 7.4) with continuous shaking at 250 rpm for 7 days. Fibrils were pelleted by centrifugation at 10,600× *g* for 15 min and suspended in sterile PBS at a concentration of 1 mg/mL. Fibril assembly was confirmed by Congo red staining. To generate the α-synuclein seeds, α-synuclein PFFs were sonicated for two cycles of 6 s on 50% power (10 microns amplitude) using a probe sonicator (Soniprep 150, Heathfield, UK) immediately before the surgical injections. A new aliquot of sonicated α-synuclein PFF was prepared every day of surgery.

## Stereotaxic surgery

Mice were deeply anesthetized with isoflurane and placed in a stereotaxic frame. Animals were unilaterally injected with 5 μL (two 2.5-μL injections) of freshly sonicated α-synuclein PFF (1 mg/mL) or PBS into the right striatum at a rate of 0.25 μL/min as previously described [15]. The coordinates of the striatum were calculated with respect to bregma using the atlas of Paxinos and Watson [22]: AP + 0.2 mm, ML − 2.0 mm, and DV − 3.4 and − 2.6 mm. After administration, the needle was left for 5 min at the injection site.

## Behavioral testing

To evaluate motor function, mice were tested using the wire hanging and the limb clasping tests. The wire hanging test was conducted before treatment, at 30-day intervals during the study and prior to sacrifice, while the limb clasping test was performed at sacrifice day. Mice were habituated to the testing room for at least 30 min before each test and behavioural tests were performed between 09:00–12:00 in the light cycle. Mouse performance was blindly evaluated by a trained observer.

### Wire hanging test

Each mouse was placed on a wire lid of a conventional housing cage. The lid was gently waving three times to ensure the grip of the mouse to the lid bars and then was turned upside down at approximately 25 cm above a surface with bedding material. The latency of mice to fall off the wire grid was recorded over a maximum period of 15 min and averaged over two trials (15 min apart).

### Negative geotaxis test

Each mouse was placed with its head facing downward on a wire grid that was set at a 45^◦^ angle to the plane. The behaviour of the animal was observed during 30 s and scored as follows: 0, turns and climbs; 1, turns and freezes; 2, moves, but fails to turn; and 3, does not move [23]. The latency to turn 180º to an upright position and initiate climbing was recorded for all animals that received a score of 0. If the mouse was unable to turn, the default value of 30 s was taken as maximal severity of impairment.

### Hind limb clasping test

Mice were held by the mid-section of the tail for 10 s. During tail suspension, limb position was scored using a 4-stage scale: 0, no change in hind limb position; 1, one hind limb retracted close to the body; 2, both hind limbs retracted close to the body; and 3, both hind limbs retracted and folded tightly along the belly.

## Enzyme-linked immunosorbent assay

On sacrifice day, mouse whole blood was collected by cardiac puncture. After resting for 60 min, supernatant containing serum was isolated from whole blood through centrifugation at 2000× *g* for 10 min. Proinflammatory cytokines in serum, including TNF-α, IFN-γ, IL-1β and IL-6, were measured using the corresponding ELISA immunoassay kit from Proteintech according to the manufacturer’s protocols.

## Western blot analysis

Cell, brain and spinal cord samples were homogenized in lysis buffer containing 10 mmol/L Tris/HCl (pH 7.4), 0.1% SDS, protease inhibitor cocktail (Thermo Scientific) and DNase (Promega) and incubated for 1 h at 37 ºC. Lysates were centrifuged at 13,000 rpm for 10 min and the supernatants were collected. Protein concentrations were determined by the BCA method. Distal intestine samples were homogenized in the same buffer containing 8 mol/L urea. Protein samples were solubilised in LDS buffer and reducing agent, separated on NuPAGE Novex 4%−12% Bis–Tris Gels (Invitrogen) and transferred to PVDF membranes. Membranes were blocked with 10% non-fat dried milk solution prepared in PBS and then incubated overnight at 4 °C with the following primary antibodies: anti-α-synuclein (Abcam, Cambridge, UK, ref# ab1903, dilution 1:2000), anti-tyrosine hydroxylase (TH) (Abcam, ref# ab112, dilution 1:5000) and anti-β-actin (Abcam, ref# ab6276, dilution 1:20,000). After wash steps, membranes were incubated with the corresponding horseradish peroxidase-conjugated secondary antibodies and immunoreactivity was detected by the luminol-based chemiluminescence (ECL) system using the ChemiDoc MP Imaging System (Bio-Rad, Hercules, CA). Densitometry analysis was performed using ImageJ software and normalized to β-actin levels.

## Immunohistochemistry and immunofluorescence

For immunohistochemistry, brain, spinal cord and intestine samples were collected after perfusion with 4% paraformaldehyde in PBS, followed by overnight postfixation, cryoprotection in 30% sucrose and freezing. Brains and spinal cords were sectioned into 30-μm coronal sections using a freezing microtome while intestines were sectioned into 12-μm transversal sections using a cryostat. Sections were washed with TBS and endogenous peroxidase activity was inactivated by incubation in 3% hydrogen peroxidase. After washing with TBS, sections were blocked in 5% normal goat serum/0.2% Triton X-100 in TBS followed by incubation overnight at 4 ºC with primary antibodies for α-synuclein (Abcam, ref# ab1903, dilution 1:2000), phospho-S129-α-synuclein (Abcam, ref# ab51253, dilution 1:2000) and TH (Abcam, ref# ab112, dilution 1:2000). For colorimetric immunostaining, sections were rinsed and incubated with biotinylated secondary antibodies of the appropriate species followed by incubation with peroxidase‐conjugated avidin. After wash steps, sections were incubated with diaminobenzidine. For immunofluorescence staining, sections were incubated with fluorescent secondary antibodies. All the samples were processed simultaneously to allow comparison.

## Image analysis

The total number of dopaminergic neurons within the SNc was assessed by stereology in every fourth section spanning the entire SNc using StereoInvestigator software (MBF Bioscience, Williston, VT) as previously described [15]. Optical density (OD) of TH immunostaining in striatum was used as an index of the density of striatal dopaminergic innervation. Briefly, nine representative sections at three coronal levels of the striatum (bregma coordinates AP: + 1.3, + 0.5 and − 0.4) were examined from each animal and pixel densities of TH-positive fiber innervation in the striatal areas were estimated using ImageJ. The obtained values were normalized by subtracting the OD of background staining measured in the white matter, where TH-positive innervation is negligible.

For assessment of α-synuclein inclusions in dopaminergic neurons, coronal sections (120 µm intervals between sections) for each animal were double-labeled using antibodies for phospho-S129 α-synuclein and TH. Numbers of phospho-α-synuclein aggregates were manually quantified at 20× magnification from regularly spaced every fourth sections throughout the rostrocaudal axis of the SNc. The data represent the total number of aggregates per section. Numbers of phospho-α-synuclein aggregates in the cortex and striatum were manually counted in one image for each section, with 3 representative sections (bregma coordinates AP: + 1.3, + 0.5 and − 0.4) for each animal. Numbers of phospho-α-synuclein aggregates in the amygdala were manually counted in one image for each section, with 2 representative sections (bregma coordinates AP: − 1.9 and − 2.2) for each animal.

## Quantitative PCR

Total RNA was isolated from frozen cell and tissue samples using the RNeasy kit (Qiagen, Hilden, Germany) according to the manufacturer’s protocol, followed by reverse transcription with the qSCRIP Reverse Transcriptase kit (Primer Design, Southampton, England) to generate cDNA. qPCR experiments were performed using the NZY Supreme qPCR Green Mastermix (Nzytech, Lisbon, Portugal) on a QuantStudio 5 Real-Time PCR system (Applied Biosystems, Waltham, MA). The primer sequences were as follows: α-synuclein, forward: 5′-GCCAAGGAGGGAGTTGTGGCTGC-3′; reverse: 5′-CTGTTGCCACACCATGCACCACTCC-3′; β-actin, forward: 5′-TCTACAATGAGCTGCGTGTG-3′; reverse: 5′-GGTGAGGATCTTCATGAGGT-3′. Expression of α-synuclein was normalized to the β-actin gene. For relative quantification, values were calculated using the comparative CT (^ΔΔ^C_t_) method.

## Statistical analysis

All statistical analyses were performed using the SPSS software version 25.0. Data are expressed as means ± standard error of the mean (SEM). Data normality was examined by the Shapiro–Wilk test. One-way analysis of variance (ANOVA) was used for comparisons between multiple groups under the assumption of normality. Tukey’s post-hoc test was used for post-hoc multiple comparisons after one-way ANOVA. For data that did not follow a normal distribution, statistical differences were analyzed by non-parametric Kruskal–Wallis test followed by the Mann–Whitney U-test. *P* < 0.05 was considered statistically significant.

## Results

### Optimization and validation of h-RVG-EVs loaded with shRNA-MCs

**Fig. 1:**
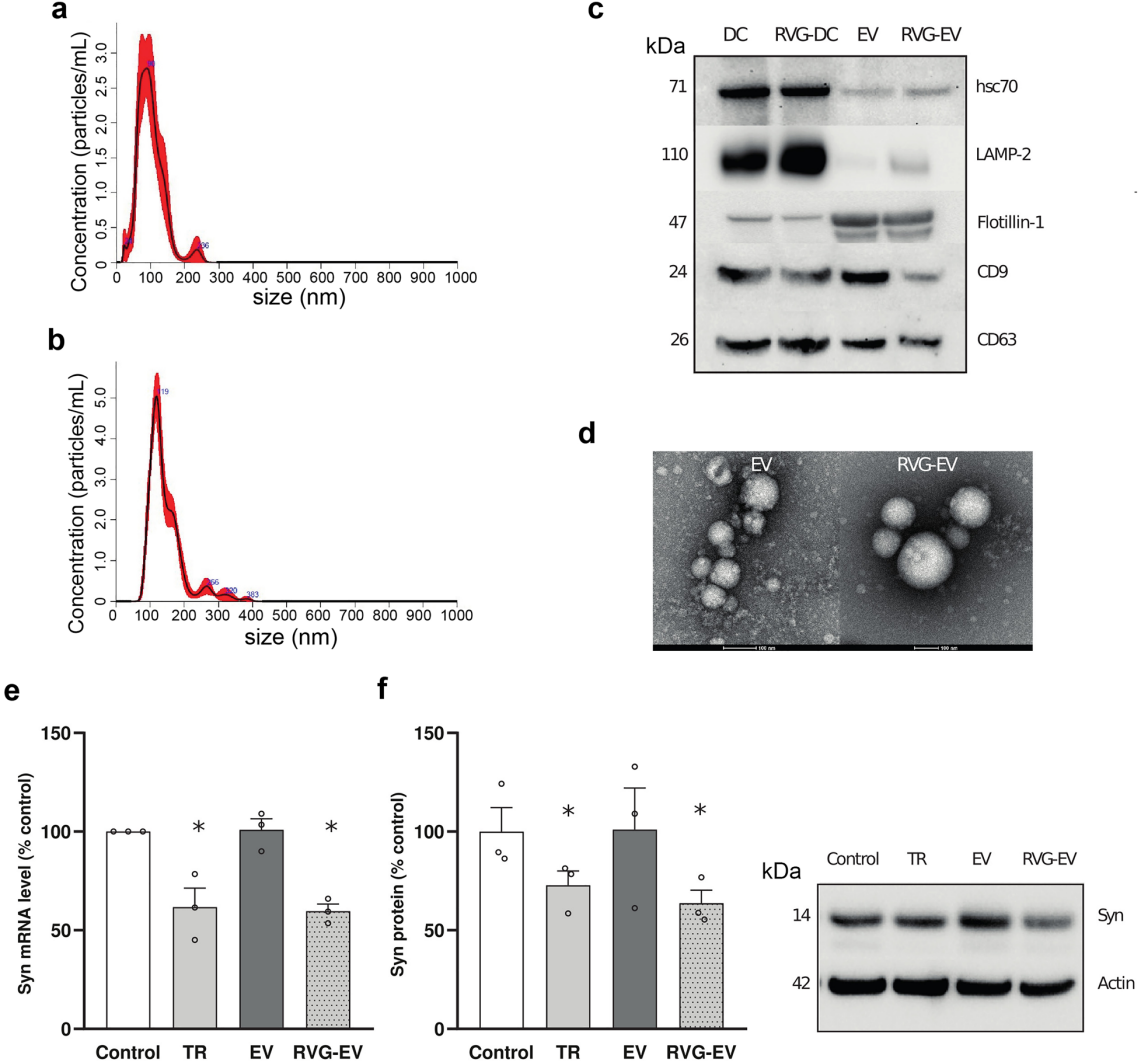
Optimization of loading and validation of h-RVG-EVs loaded with anti-α-synuclein shRNA-MCs. **a**, **b** Nanoparticle tracking analysis characterization of EVs isolated from control hDCs (**a)** and hDCs electroporated with hLamp2b-RVG mRNA (**b**). **c** Western blot analysis to assess expression of markers in control EV and RVG-EV. **d** Representative scanning electron microscopy images of EV isolated from control hDCs and hDCs electroporated with hLamp2b-RVG mRNA. Scale bar, 200 nm. **e**, **f** SH-SY5Y cells overexpressing α-synuclein were treated with anti α-synuclein shRNA-MC using a transfection reaction (TR), or loaded into control h-EVs (EV) and h-RVG-EV (RVG-EV). α-synuclein mRNA (**e**) and protein (**f**) levels were quantified and normalized to control cells. Typical western blots are shown. Data are expressed as mean ± SEM (*n* = 4). *P < 0.05, non-parametric Kruskal–Wallis test, statistical analyses compared to untreated control cells

To produce brain-targeted EVs, human Lamp2b (hLamp2b) protein was fused with the RVG peptide. In previous studies using mouse DCs as a source of RVG-EVs, the DCs were transfected with plasmids encoding mouse Lamp2b-RVG construct. However, hDCs are extremely difficult to transfect with plasmids. Alternatively, hLamp2b-RVG mRNA was synthetized in vitro and hDCs were electroporated with the mRNA. Optimization using GFP mRNA confirmed a transfection efficiency of 95%. Detection of specific markers of hDCs including CD80, CD86 and MHC-II was performed by Western blot and morphology of hDCs was confirmed by microscopy (Fig. S1). EVs isolated from control hDCs and hDCs electroporated with hLamp2b-RVG mRNA were characterized by nanoparticle tracking analysis (Fig. 1a, b), Western blot (Fig. 1c) and transmission electron microscopy (Fig. 1d) following the Minimal Information for Studies of EV (MISEV) guidelines [24, 25]. The control EVs and RVG-EVs had similar sizes and protein compositions. Subsequently, h-RVG-EVs (5 × 10^8^) were loaded with syn-MCs (1 mg) using conditions previously optimized for Ms-RVG-EVs [15]. To analyze the downregulation efficiency of the h-RVG-EVs loaded with syn-MCs, SH-SY5Y cells over-expressing wild-type α-synuclein were treated with control hEVs or h-RVG-EVs loaded with syn-MCs. As a positive control, syn-MCs were introduced into the SH-SY5Y cells using a commercial transfection reagent. Seventy-two hours after treatment, α-synuclein mRNA and protein levels were decreased by 41% (*P* = 0.037) and 37% (*P* = 0.046), respectively, in cells treated with syn-MC-loaded h-RVG-EVs (Fig. 1e, f). The mRNA and protein levels were decreased to a similar extent using a transfection reagent (decreased 39%, *P* = 0.037 and 28%, *P* = 0.049, respectively) (Fig. 1e, f). However, in SH-SY5Y cells treated with unmodified EVs loaded with syn-MCs, the α-synuclein mRNA and protein levels were unaffected (Fig. 1e, f).

## shRNA-MC h-RVG-EVs reduce α-synuclein levels and pathology in the brain

To investigate the distribution of h-RVG-EVs after systemic delivery, h-RVG-EVs labeled with DiR were injected iv into normal mice (Supplementary Methods). Twenty-four hours after injection, the strongest signal was detected in the liver and the spleen, with detectable levels in the kidneys, heart, lungs, intestine and brain (Fig. S2). To assess the therapeutic effect of the treatment, the progressive α-synuclein PFF mouse model [19] received two iv injections (via the tail vein) of h-RVG-EVs loaded with syn-MCs (*n* = 18), h-RVG-EVs loaded with anti-GFP shRNA-MCs (GFP-MCs) (*n* = 18), or vehicle (5% glucose; *n* = 18) on days 2 and 45 after PFF injection. An additional group of α-synuclein PFF injected mice received two iv injections of Ms-RVG-EVs loaded with syn-MCs (*n* = 12). Control animals were injected with sterile PBS in the striatum and received two iv injections of vehicle (*n* = 18). Mice were sacrificed 90 days after PFF injection.

**Fig. 2:**
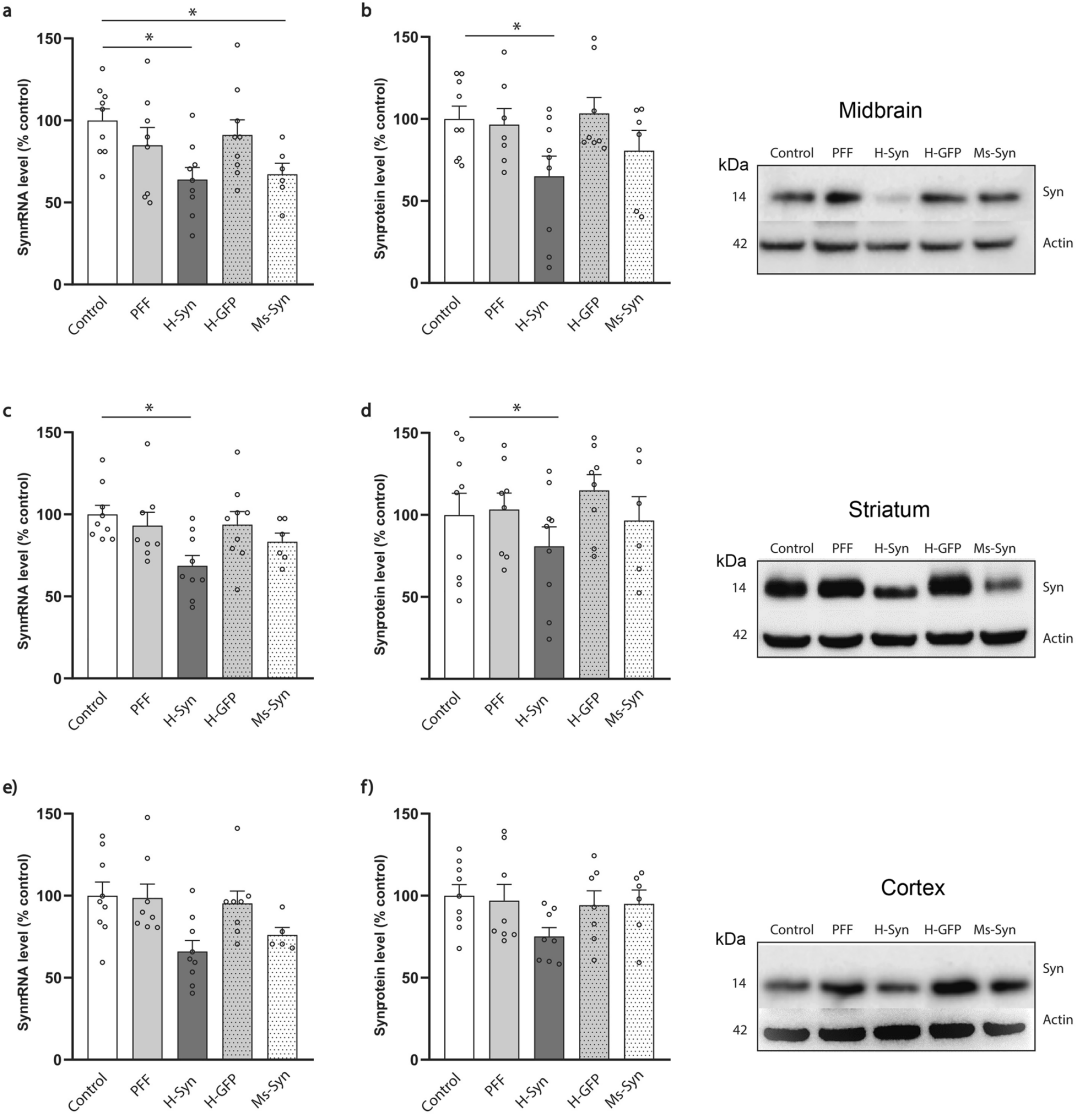
α-synuclein downregulation in several brain regions of mice treated with syn-MCs delivered by h-RVG-EVs. Analyses of α-synuclein mRNA expression (**a**, **c**, **e**) and protein (**b**, **d**, **f**) levels normalized to actin in the ipsilateral midbrain (**a**, **b**), striatum (**c**, **d**) and cortex (**e**, **f**) of α-synuclein PFF-treated mice (PFF) after treatment with h-RVG-EVs loaded with anti-α-synuclein (H-Syn) or GFP (H-GFP) shRNA-MCs. A group of mice was treated with anti-α-synuclein shRNA-MCs delivered by Ms-RVG-EV (Ms-Syn). Typical western blots are shown. Data are expressed as mean ± SEM (*n* = 6–8). *P < 0.05 compared to control mice, one-way ANOVA

qPCR analysis confirmed that α-synuclein mRNA expression was downregulated in both ipsilateral (decreased 36%, *P* = 0.027, Fig. 2a) and contralateral midbrain (decreased 40%, *P* = 0.024, Fig. S3a) of mice treated with h-RVG-EVs containing syn-MCs. The long-term (90 days) down-regulation of α-synuclein mRNA levels in midbrain was associated with lower levels of α-synuclein protein in this region (ipsilateral: decreased 35%,* P* = 0.045 Fig. 2b; contralateral: decreased 40%, *P* = 0.0039, Fig. S3). The α-synuclein mRNA expression was also decreased in the ipsilateral striatum (decreased 32%, *P* = 0.0021) and cortex (decreased 35%, *P* = 0.017) of mice treated with syn-MC h-RVG-EV therapy, associated with a concomitant reduction in α-synuclein protein levels in both brain regions (striatum: decreased 20%, *P* = 0.049; cortex: decreased 25%, not significant) (Fig. 2c–f). However, in animals treated with h-RVG-EVs loaded with GFP-MCs, α-synuclein mRNA and protein levels remained similar to controls and vehicle-treated PFF mice, confirming the specificity of syn-MC-induced down-regulation (Fig. 2a–f, Fig. S3). The injection of syn-MCs loaded into Ms-RVG-EVs significantly decreased mRNA expression of α-synuclein only in the midbrain (ipsilateral, decreased 33%, *P* = 0.049; contralateral, decreased 34%, *P* = 0.037) (Fig. 2a–f, Fig. S3), while the protein level was not significantly reduced in any of the regions examined (Fig. 2a–f, Fig. S3).

**Fig. 3:**
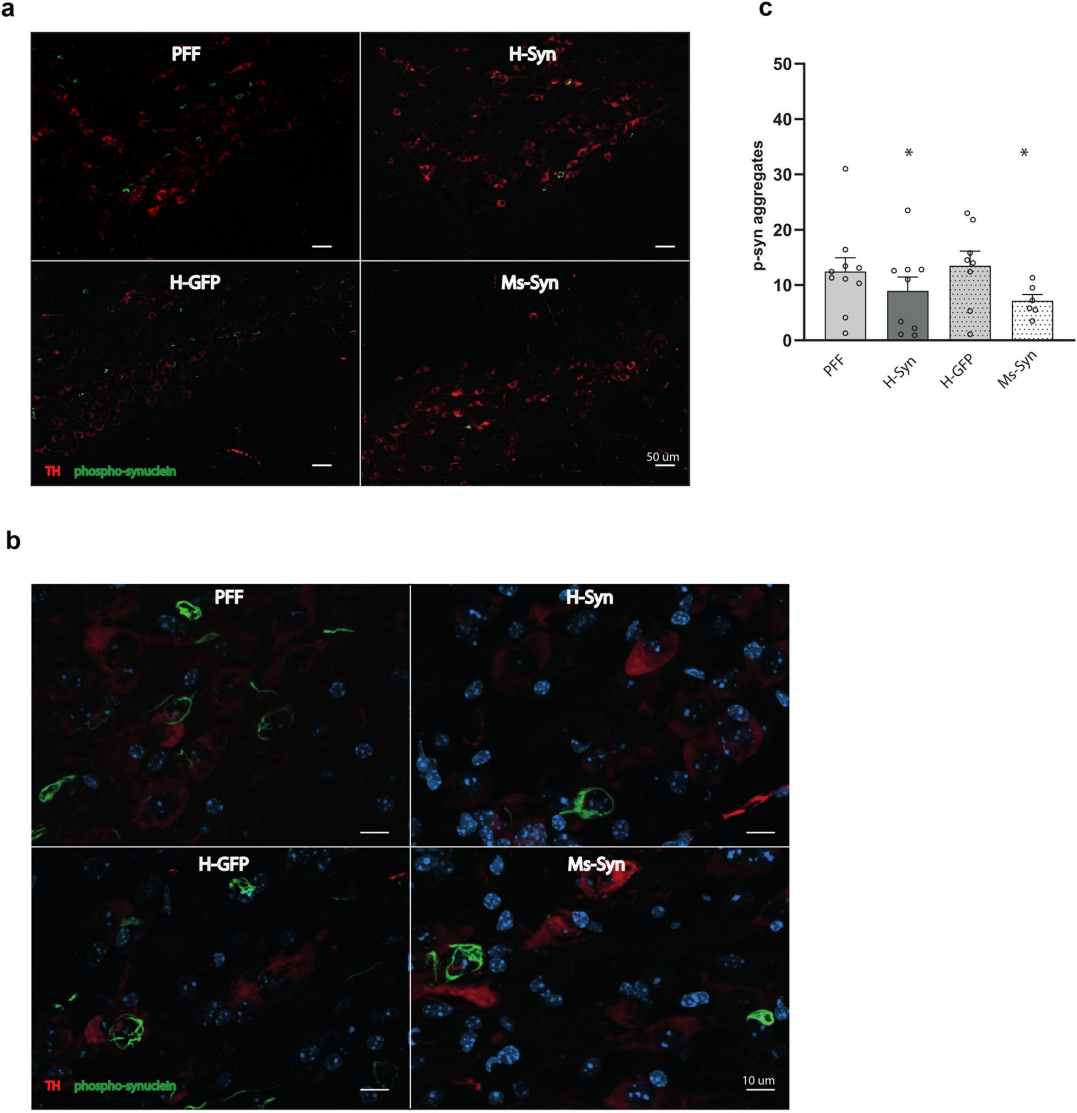
Effect of h-RVG-EVs loaded with syn-MCs on α-synuclein pathology in SNc. **a** Immunofluorescent images of midbrain sections stained with antibodies to S129 phospho-α-synuclein (green) and TH (red). Scale bar, 50 μm. **b** Magnified images of stained midbrain section showing co-localization of α-synuclein aggregates (green) in dopaminergic neurons (red). Scale bar, 10 μm. **c** Quantitation of α-synuclein-positive aggregates per section in the ipsilateral SNc of α-synuclein PFF-treated mice following treatment with h-RVG-EVs loaded with anti-α-synuclein (H-Syn) or GFP (H-GFP) shRNA-MCs or after treatment with Ms-RVG-EVs loaded with anti-α-synuclein shRNA-MCs (Ms-Syn). Data are expressed as mean ± SEM (*n* = 6–10). *P < 0.05 compared to PFF mice, one-way ANOVA

**Fig. 4:**
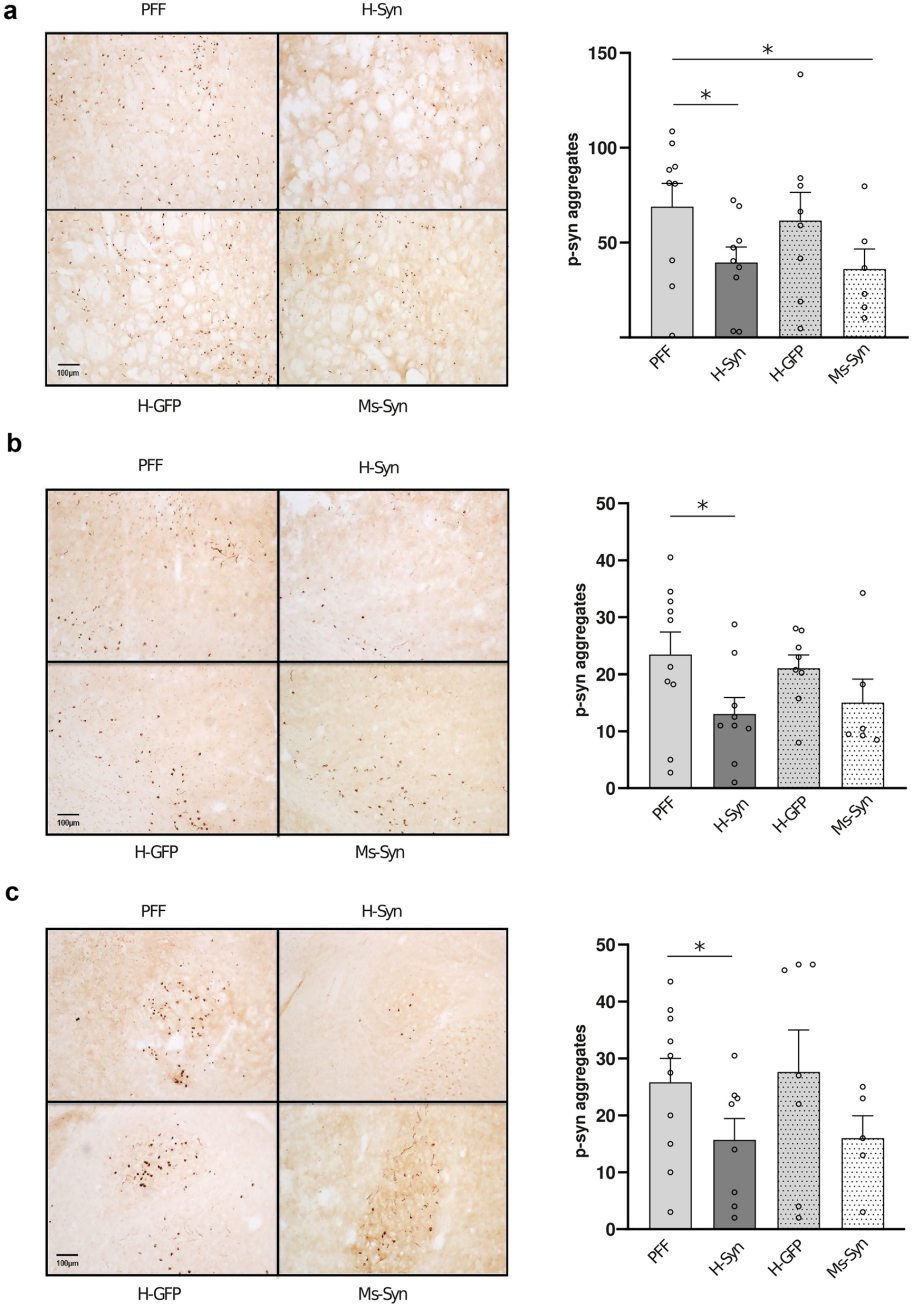
α-Synuclein pathology in the striatum, cortex and amygdala after treatment with h-RVG-EVs loaded with syn-MCs. Quantification of aggregates in the ipsilateral striatum (**a**), cortex (**b**) and amygdala (**c**) of α-synuclein PFF-injected mice (PFF) following treatment with h-RVG-EVs loaded with anti-α-synuclein (H-Syn) or GFP (H-GFP) shRNA-MCs or after treatment with anti-α-synuclein shRNA-MCs loaded in Ms-RVG-EVs (Ms-Syn). Representative immunohistochemical images of intraneuronal phospho-α-synuclein-positive aggregates in the ipsilateral striatum, cortex and amygdala are shown. Scale bar, 100 µm. Data are expressed as mean ± SEM (*n* = 6–10), *P < 0.05, one-way ANOVA compared to PFF mice

We further evaluated the impact of h-RVG-EVs loaded with syn-MCs on α-synuclein pathology. Ninety days after α-synuclein PFF injection, immunohistological staining for phospho-α-synuclein revealed abundant α-synuclein aggregates in the ipsilateral SNc of PFF-injected mice and PFF-injected mice treated with GFP-MC-loaded h-RVG-EVs (Fig. 3a, b). Most of the inclusions were localized in dopaminergic neurons (Fig. 3b). Treatment with syn-MC h-RVG-EVs significantly reduced the number of α-synuclein aggregates in the SNc (decreased 29%, *P* = 0.039), similar to the reduction observed in mice treated with Ms-RVG-EVs (decreased 37%, *P* = 0.027) (Fig. 3a, c). Similar results were found in other brain regions analyzed after treatment with syn-MC-loaded h-RVG-EVs or Ms-RVG-EVs, including striatum (h-RVG-EV decreased 43%, *P* = 0.039; Ms-RVG-EV decreased 49%, *P* = 0.038), cortex (h-RVG-EV decreased 46%, *P* = 0.033; Ms-RVG-EV decreased 35%, *P* = 0.047) and amygdala (h-RVG-EV decreased 37%, *P* = 0.043; Ms-RVG-EV decreased 36%, *P* = 0.041) (Fig. 4a–c).

## shRNA-MC-loaded h-RVG-EVs prevent neuronal degeneration and behavioral deficits

**Fig. 5:**
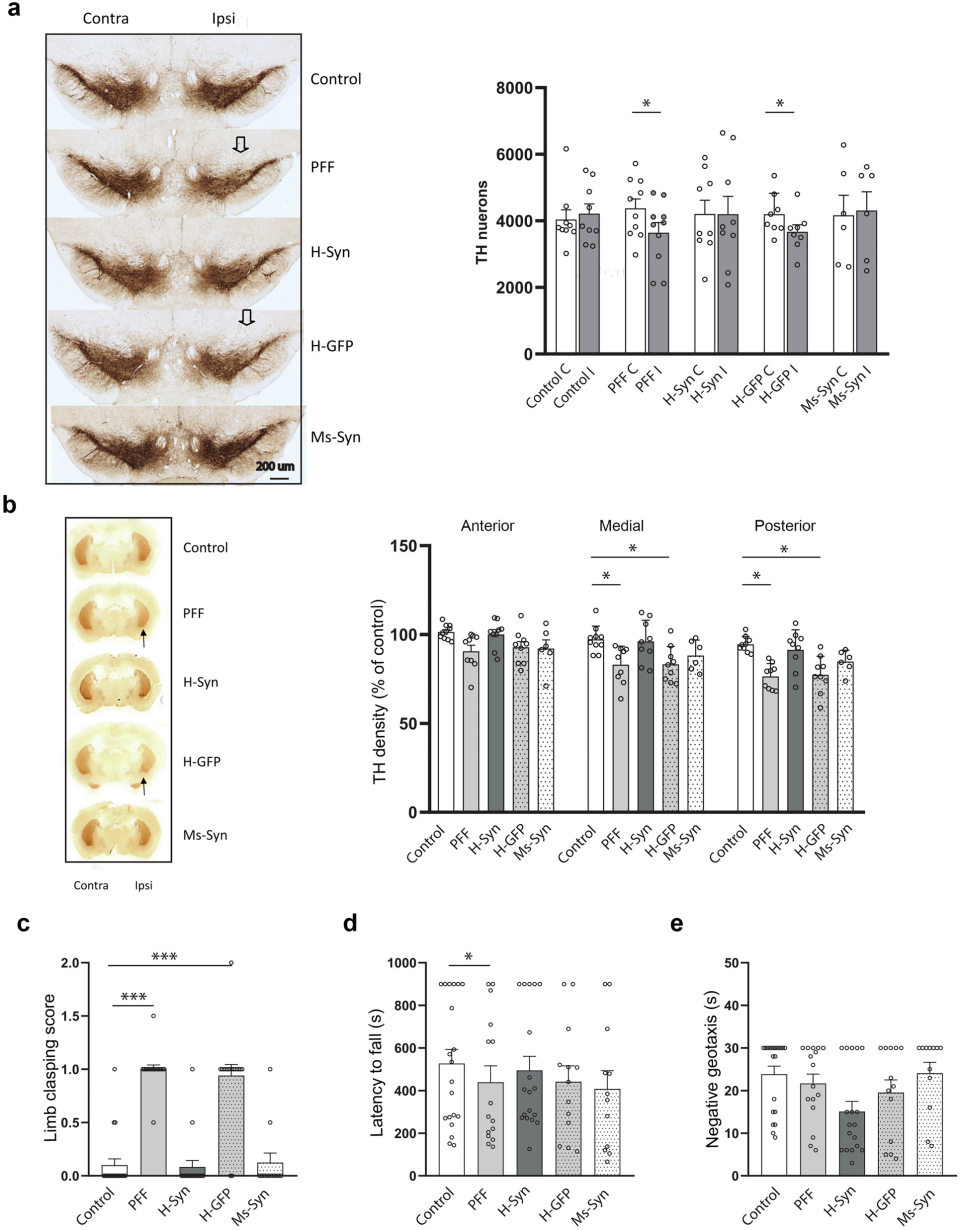
Syn-MC h-RVG-EV therapy prevented dopaminergic dysfunction and motor impairments. **a** TH staining of dopaminergic neurons in coronal midbrain sections of α-synuclein PFF-injected mice (PFF) following treatment with h-RVG-EVs loaded with anti-α-synuclein (H-Syn) or GFP (H-GFP) shRNA-MCs or after treatment with anti-α-synuclein shRNA-MCs loaded in Ms-RVG-EVs (Ms-Syn). Arrows point to area of decreased dopaminergic neurons. Numbers of nigral dopaminergic neurons were quantified by unbiased stereology on each brain hemisphere (I, ipsilateral; C, contralateral). **b** TH quantitation by optical density in ipsilateral sections of anterior, medial and posterior striatum normalized to contralateral striatum. Representative striatal section showing TH staining are shown. **c** Quantitative analysis of the hind limb clasping scores. **d** Wire hanging performance. **e** Time to turn around and move up toward the top of the platform in the negative geotaxis test. If the mouse was unable to turn, the default value of 30 s was taken as maximal severity of impairment. Data are expressed as mean ± SEM (*n* = 6–10). *P < 0.05, ***P < 0.001 compared to control mice, one-way ANOVA

The presence of α-synuclein aggregates in the SNc was associated with a decreased survival of dopaminergic neurons over time. Stereological evaluation of TH-positive neurons revealed a unilateral 16% loss of dopaminergic neurons in the ipsilateral SNc 90 days after PFF injection (*P* = 0.047) (Fig. 5a). This was confirmed by western blotting for TH protein in midbrain samples (decreased 25%, non-significant) (Fig. S4). Dopaminergic degeneration was associated with a unilateral loss of dopaminergic striatal innervation reflected by a significant decrease in TH staining in medial (17% decrease compared to control, *P* = 0.016) and posterior (25% decrease compared to control, *P* = 0.000) striatum (Fig. 5b). Similarly, the PFF-injected mice treated with GFP-MC-loaded h-RVG-EVs also showed a loss of dopaminergic neurons in the SNc (14% decrease, *P* = 0.049) (Fig. 5a) and a significant decrease of TH staining in the medial and posterior striatum (17%, *P* = 0.015; 23%, *P* = 0.001, respectively).

Treatment with syn-MC-loaded h-RVG-EVs prevented the dopaminergic neuronal degeneration (Fig. 5a) and this protective effect was associated with a significant preservation of dopaminergic innervation in the striatum (Fig. 5b). Similar results were observed from treatment with Ms-RVG-EVs loaded with syn-MCs (Fig. 5a, b).

Consistent with previous studies, dopaminergic dysfunction significantly compromised motor performance of mice in the wire hanging (*P* = 0.039) and the limb clasping tests (*P* = 0.000) (Fig. 5c, d). Similar results were observed in PPF mice treated with GFP-MC-loaded h-RVG-EVs (Fig. 5c, d). However, the syn-MC h-RVG-EV therapy improved the motor performance, which was comparable to the performance of control mice (Fig. 5c, d). The PFF mice treated with Ms-RVG-EVs performed similar to the control group in the limb clasping test (Fig. 5c), although an impairment in the wire hanging test performance was observed (Fig. 5d). There were no significant differences between the 5 groups in the performance in the negative geotaxis test (Fig. 5e).

Overall, these results demonstrate that syn-MC h-RVG-EVs can prevent dopaminergic neuronal degeneration and motor impairments in the α-synuclein PFF mouse model of PD.

## Effects of shRNA-MC-loaded h-RVG-EVs on olfactory bulb, spinal cord and intestine

**Fig. 6:**
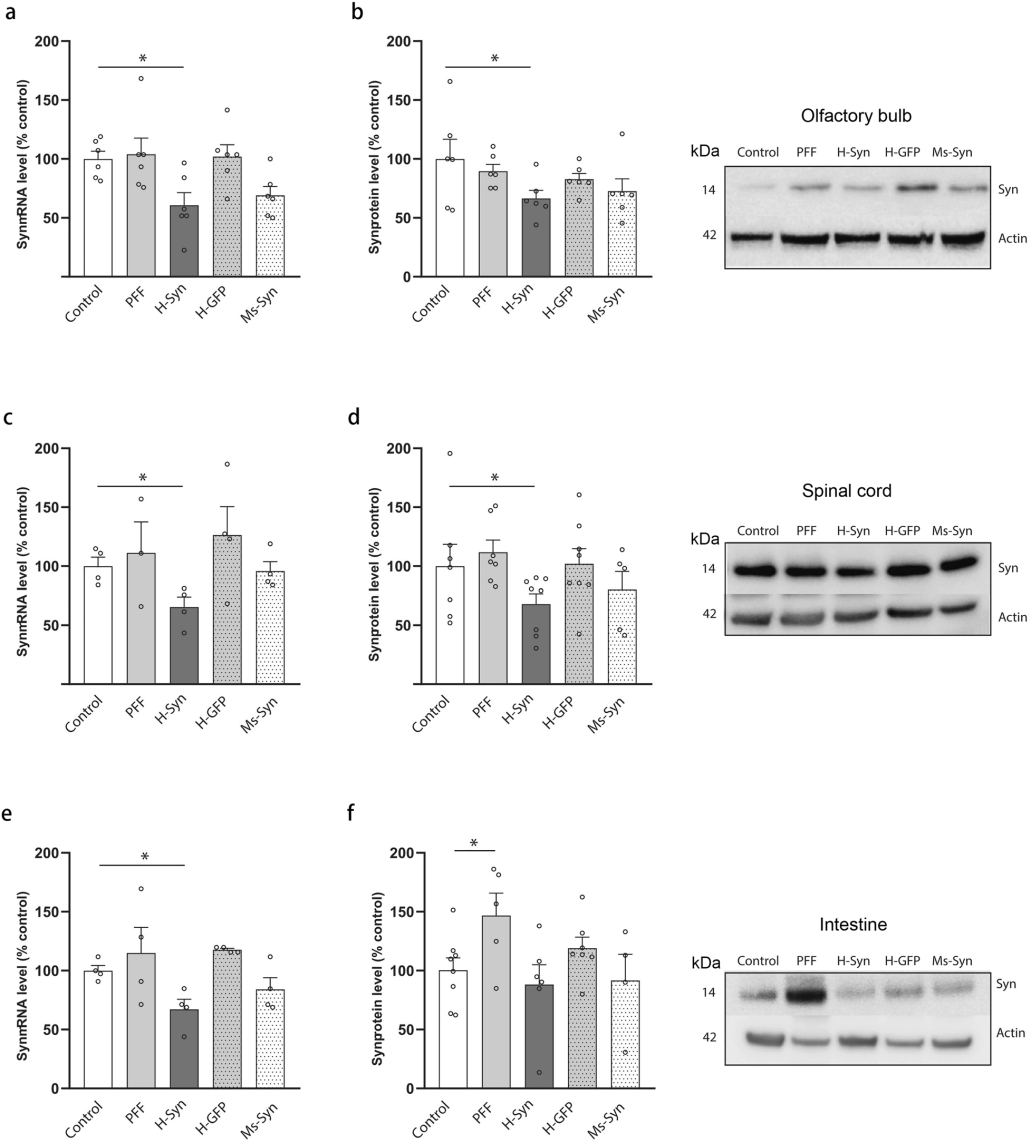
Effect on α-synuclein mRNA and protein levels in the spinal cord, intestine and olfactory bulb. Quantitation of α-synuclein mRNA (**a**, **c, e**) and protein (**b**, **d, f**) levels in the olfactory bulb (**a** and **b**), spinal cord (**c** and **d**) and intestine (**e** and **f**) from α-synuclein PFF-treated mice (PFF) at 90 days following treatment with h-RVG-EVs loaded with anti-α-synuclein (H-Syn) or GFP (H-GFP) shRNA-MCs or after treatment with anti-α-synuclein shRNA-MCs loaded in Ms-RVG-EVs (Ms-Syn). Typical western blots are shown. Data are expressed as mean ± SEM (*n* = 6–8). *P < 0.05 compared to control mice, one-way ANOVA

To investigate if h-RVG-EVs loaded with syn-MCs can down-regulate α-synuclein expression in brain regions or organs affected at early stage of the disease, mRNA expression and protein level of α-synuclein were analyzed in the olfactory bulb, spinal cord and intestine samples. Treatment with syn-MC h-RVG-EVs significantly reduced α-synuclein mRNA expression (decreased 39% compared to controls, *P* = 0.016) (Fig. 6a) and protein level in the olfactory bulb (decreased 34% compared to controls, *P* = 0.060) (Fig. 6b). A previous study demonstrated that intrastriatal injection of α-synuclein PFF is associated with α-synuclein inclusions in the spinal cord and the intestine [26]. In this study, immunohistochemical analyses confirmed that h-RVG-EVs loaded with syn-MCs prevented the α-synuclein aggregate formation in the spinal cord (Fig. S5a) and the effect was associated with decreased mRNA expression (decreased 35% compared to controls, *P* = 0.021) (Fig. 6c) and protein level (decreased 32% compared to controls, *P* = 0.021) of α-synuclein (Fig. 6d). However, Ms-RVG-EVs loaded with syn-MCs or h-RVG-EVs loaded with GFP-MCs did not significantly alter the mRNA expression and protein level of α-synuclein (Fig. 6c, d). A similar decrease in α-synuclein mRNA expression (decreased 33% compared to controls, *P* = 0.021), protein level (decreased 15% compared to controls, non-significant) and pathology was found in the distal intestine of PFF mice treated with syn-MC-loaded h-RVG-EVs (Fig. 6e, f and Fig. S5b, c).

Finally, to exclude any detrimental effect of h-RVG-EVs loaded with syn-MCs, transcriptomic analysis of contralateral cortical samples was performed (Supplementary Methods). Results demonstrated no changes in mRNA expression in PFF mice treated with vehicle, h-RVG-EVs loaded with syn-MCs, or h-RVG-EVs loaded with GFP-MCs compared with controls (Table S1). Moreover, no inflammatory response was activated as the levels of TNF-α, IFN-ɣ, IL-4 and IL-1β were unaffected (Table S2).

## Proteomic analysis of RVG-EVs from control and PD patient donors

**Fig. 7:**
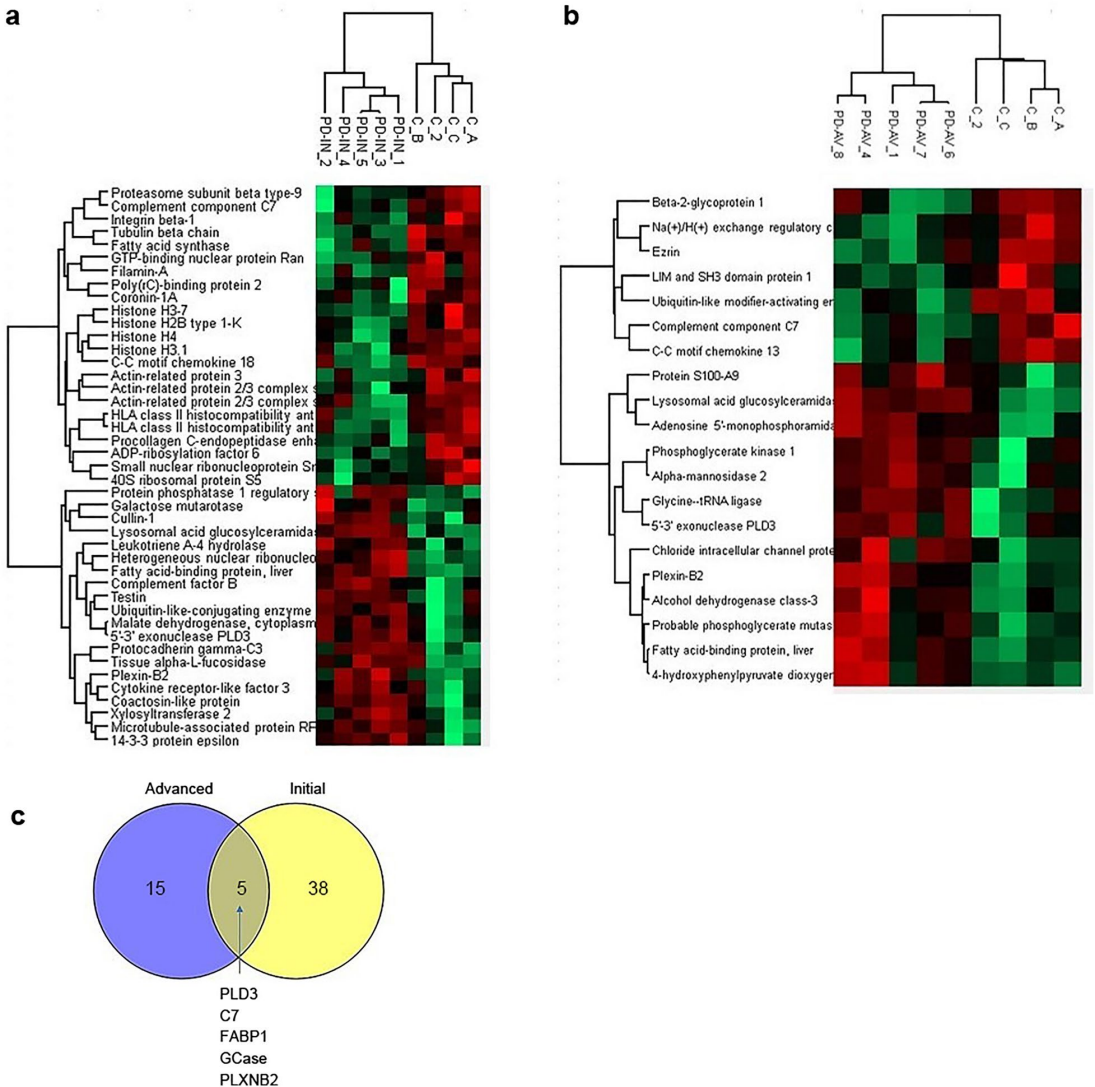
Proteomic analysis of EVs produced by DCs isolated from control, initial PD and advance PD. **a, b** Heat map analysis showing differentially expressed proteins between initial PD (**a)** or advanced PD (**b**) and control patients. **c** Venn diagram of differentially expressed proteins detected in EVs derived from initial and advanced PD

The implementation of the therapy requires development of a source of targeted EVs compatible with a personalized therapy; this implies the development of a self-cell source for derivation of immunologically inert EVs. To confirm that EVs derived from PD patients are suitable vehicles, proteomic analysis of EVs derived from control, initial PD and advanced PD was performed. The proteomic analysis confirmed that EVs produced by DCs differentiated in vitro from blood monocytes from PD patients were suitable as therapeutic vehicles. A total of 756 proteins were identified, and differential expression analysis showed minimal changes in protein abundance (Fig. 7a, b). Proteomic analysis did not detect α-synuclein in EVs isolated from hDCs of healthy controls, initial PD patients or advance PD patients, supporting the absence of α-synuclein in EVs. Expression of 43 proteins was altered in initial PD EVs compared to controls, while 20 proteins were differentially expressed in advanced PD EVs compared to control EVs. Five proteins were consistently altered in EVs derived from DCs of patients with initial and advanced PD (Fig. 7c). Remarkably, one protein was lysosomal acid glucosylceramidase (GCase), which is related with familial PD and altered in sporadic PD.

## Discussion

The development of PD therapies faces two major challenges, the presence of the blood–brain barrier and the need to administer multiple doses since PD is a long-term condition. To solve these issues, we have adapted a therapeutic approach previously developed by our group using mouse targeted EVs to deliver MCs expressing shRNA to induce long-term α-synuclein down-regulation [15]. Although the previous therapy successfully reduced the pathology, and prevented dopaminergic cell death and motor abnormalities, the clinical translation is extremely unlikely since they are mouse-derived EVs. To overcome this problem, we have developed a source of human RVG-EVs compatible with a personalized therapy using immature hDCs. EVs derived from immature hDCs minimize the possibility of an immune response, allowing long-term treatment. We generated the immature hDCs in vitro by differentiating peripheral blood monocytes isolated from discarded blood donation fractions. However, for clinical application, monocytes would be isolated by apheresis from patients themselves, a strategy previously used successfully in clinical trials in oncologic patients [20, 21].

Regarding therapeutic efficiency, this study demonstrated that syn-MCs delivered by h-RVG-EVs reduced α-synuclein mRNA expression and protein level in a greater proportion than shRNA-MCs delivered by Ms-RVG-EVs. Although the two treatments showed similar reduction of α-synuclein aggregates and protection of dopaminergic neurons, animals treated with shRNA-MCs delivered by Ms-RVG-EVs showed a unilateral loss of dopaminergic innervation associated with motor impairment. A clear advantage of this approach compared to other therapies is the ability of human RVG-EVs to distribute and deliver their cargoes throughout the brain after an iv injection, which simplifies the treatment administration, and potentially could prevent or decrease PD progression. Moreover, the lack of immune activation and off-target effects confirmed by transcriptomic analysis supports the use of this approach as a safe multi-dose therapy required for long-term treatments. As previously described for mouse RVG-EVs [15], the dose of syn-MCs tested allows downregulation of α-synuclein levels that is capable of decreasing the pathology but sufficient to maintain the normal function of α-synuclein. In vivo studies demonstrated that the level of α-synuclein downregulation is critical for avoiding any detrimental effects of the treatment. A decrease of 45%–85% in the brain achieved in some studies was associated with a 25%–50% reduction of dopaminergic neurons and an inflammatory response [27, 28]. However, milder reduction of α-synuclein in different PD mouse models described in other studies, protected against the pathological neurodegenerative process and had no detrimental effects on dopaminergic neurons [15, 29, 30].

These results reinforced the therapeutic potential of shRNA-MCs delivered by h-RVG-EVs to treat PD and other synucleinopathies. Finally, the reductions of α-synuclein levels in other structures such as the olfactory bulb, intestine and spinal cord indicate the potential use of the treatment in early phases of PD. Treatment at PD initial stages is an increasingly closer possibility due to the development of new diagnostic biomarkers based on the detection of pathological α-synuclein [31].

The lack of detection of α-synuclein in EVs isolated from DCs of PD patients excludes the potential spreading of pathological α-synuclein within EVs to other regions. Some studies have reported changes in monocytic populations [32, 33] and transcriptomic alterations [34] in PD peripheral monocytes. Since our approach plans to use DCs differentiated in vitro from self-blood monocytes as a source of RVG-EVs, we also assessed the composition of EVs derived from control and PD monocytes. Proteomic analysis of EVs isolated from control and PD patient DCs revealed a very similar composition with few proteins differentially expressed, confirming that EVs from PD patients are suitable as a personalized therapeutic vehicle. Interestingly, one of the proteins enriched in PD EVs was GCase, the most important genetic risk factor in PD and a protein with lower levels and decreased activity in sporadic PD brain samples [35]. A recent report demonstrated a reduction in GCase activity in PD monocytes with normal protein levels [36]; however, our data showed an increase in GCase levels in EVs produced by hDCs differentiated from PD blood monocytes.

Early phase clinical trials with DCs and EVs derived from mesenchymal stem cells showed the safety of EVs and to some extent the feasibility of clinical-scale EV production [20, 21]. There have also been reports of rapid large-scale purification of cell culture-derived therapeutic EVs [37, 38]. These advances support the development of GMP-grade shRNA-MC h-RVG-EV therapies that could be tested in clinical trials. This implies the transfer of h-RVG-EV generation and loading to GMP processes and the preclinical evaluation of the shRNA-MC h-RVG-EV GMP grade.

To our knowledge, we described for the first time the use of human EVs loaded with shRNA-MCs for gene therapy. We reported the development of a novel gene therapy delivered using human targeted EVs for a neurodegenerative disease that is compatible with a personalized therapy and could be generated using GMP processes. These results open a new avenue for the treatment of PD and a wide variety of other neurological diseases including Alzheimer’s disease, Huntington’s disease, amyotrophic lateral sclerosis and prion disease, for which efficacious treatments are currently lacking. In the future, this technology could be adapted for different drug targets and the h-RVG-EVs could deliver other molecules including siRNAs, miRNAs, ASOs and proteins. Successful development and clinical implementation of this technology has the potential to benefit sufferers of various diseases as well as their families and caregivers.

## Conclusions

Our study confirmed that shRNA-MCs delivered by h-RVG-EVs decrease α-synuclein level and aggregation and reduce dopaminergic cell death. Our results support the feasibility of this minimally invasive disease-modifying therapy that is compatible with a personalized therapy. These results reinforce the therapeutic potential of shRNA-MCs delivered by h-RVG-EVs to treat PD and other synucleinopathies.

## Supplementary Information

**Additional file 1. Supplementary Methods**.** Fig. S1**. Human dendritic cells characterization. **Fig. S2**. In vivo organ biodistribution of human RVG-EVs. **Fig. S3**. Alpha-synuclein downregulation in contralateral brain regions of mice treated with anti-alpha-synuclein shRNA-MC delivered by h-RVG-EV. **Fig. S4**. Quantification of TH protein levels in midbrain. **Fig. S5**. Immunofluorescence images of total alpha-synuclein in cord and intestinal sections and immunohistochemical images of phosphorylated alpha-synuclein in intestine. **Table S1**. Changes in gene expression. **Table S2**. Inflammatory cytokine levels in PFFs alpha-synuclein treated mice analyzed 90 days after the first treatment. **Uncropped Western blots.**

## Data Availability

All data associated with this study are present in the paper or the Supplementary Materials. Mass-spectrometry data and search results files were deposited in the Proteome Xchange Consortium via the JPOST partner repository (https://repository.jpostdb.org) with the identifier PXD051578 for ProteomeXchange and JPST003045 for jPOST (for reviewers: https://repository.jpostdb.org/preview/182047976366227b5d37f67 Access key: 2026).

## Abbreviations

EV: Extracellular vesicles
hDCs: Human dendritic cells
MC: Minicircle
PBMCs: Peripheral blood mononuclear cells
PD: Parkinson’s disease
PFF: Preformed fibrils
RVG: Rabies virus glycoprotein
SNc: Substantia nigra pars compacta
TH: Tyrosine hydroxylase
