# Supplementary material for: Development of human targeted extracellular vesicles loaded with shRNA minicircles to prevent parkinsonian pathology
Source: Transl Neurodegener. 2025 May 26;14:26. doi: 10.1186/s40035-025-00484-7 (PMC12105355; doi:10.1186/s40035-025-00484-7)

SUPPLEMENTARY METHODS:

Transcriptomic analysis:

The quality of the RNASeq results was assessed using FastQC (v0.12.1; https://www.bioinformatics.babraham.ac.uk/projects/fastqc/). The raw rads were trimmed, filtered with a Phred quality score of at least 25 and all adapters removed with FastP (v0.23.4) software. Clean reds were aligned versus the Mus Musculus reference genome (release GRCm39_109, https://ftp.ensembl.org/pub/release-109/fasta/mus_musculus/dna/) using HiSat2 (v2.2.1, https://daehwankimlab.github.io/hisat2/) with default parameters. Resulting alignment files were quality assessed with Qualimap2 (v2.2.1, https://www.ncbi.nlm.nih.gov/pmc/articles/PMC4708105/) and sorted and indexed with Samtools (v1.10) software. Quantitative differential expression analysis between conditions was performed both by DESeq2 (v1.34.0) and edgeR (v3.36.0) implementations, to compare paired groups. Both methodological analyses, implemented as R Bioconductor packages, perform read-count normalization following a negative binomial distribution model. In order to automate this process and facilitate all group combination analysis, the SARTools pipeline (v1.8.1) was used. All resultant data was obtained as .html files and .csv tables, including density count distribution analysis, pairwise scatter plots, cluster dendrogram, Principal Component Analysis (PCA) plots, size factor estimations, dispersion plots and MA- and volcano plots. Resulting tables, including Ensembl Gene ID, raw counts, normalized counts, Fold-Change estimation and dispersion data for each of the analysis methods (DESeq2 and edgeR) were annotated with additional data from Biomart database.

In order to control the False Discovery Rate (FDR), p-values were amended by Benjamini-Hochberg (BH) multiple testing correction. Those features showing corrected p-values below 0.05 threshold were considered up- or down-regulated genes. In order to reinforce downstream analysis and discard false-positive over/under-expressed genes, common up- and down-regulated features were extracted from DESeq2 and edgeR tables.

Proteomic analysis

Sample preparation

Protein extracts were diluted in Laemmli sample buffer and loaded into a 1.5 mm thick polyacrylamide gel with a 4% stacking gel casted over a 12.5% resolving gel. The run was stopped as soon as the front entered 3 mm into the resolving gel so that the whole proteome became concentrated in the stacking/resolving gel interface. Bands were stained with Coomassie Brilliant Blue, excised from the gel and protein enzymatic cleavage was carried out with trypsin (Promega; 1:20, w/w) at 37 °C for 16 h as previously described (Shevchenko, A. et al. In Nat Protoc 1 (6), 2856 (2006). Purification and concentration of peptides was performed using C18 Zip Tip Solid Phase Extraction (Millipore).

Data independent acquisition (DIA)-mass spectrometry

Dried down peptide samples were reconstituted with 2% ACN-0.1% FA (Acetonitrile-Formic acid), spiked with internal retention time peptide standards (iRT, Biognosys), and quantified by NanoDropTM spectrophometer (ThermoFisher Sci.) prior to LC-MS/MS analysis using an EASY-1000 nanoLC system coupled to an EZ-Exploris 480 mass spectrometer (Thermo Fisher Sci.). Peptides were resolved using C18 Aurora column (75µm x 25cm, 1.6 µm particles; IonOpticks) at a flow rate of 300 nL/min using a 60-min gradient (50 oC): 2% to 5% B in 1 min, 5% to 20% B in 48 min, 20% to 32% B in 12 min, and 32% to 95% B in 1 min (A = FA, 0.1%; B = 100% ACN:0.1% FA). Peptides were ionized using 1.6 kV spray voltage at a capillary temperature of 275 °C. Sample data were acquired in data-independent acquisition (DIA) mode with full MS scans (scan range: 400 to 900 m/z; resolution: 60,000; maximum injection time: 22 ms; normalized AGC target: 300%) and 24 periodical MS/MS segments applying 20 Th isolation windows (0.5 Th overlap: Resolution: 15000; maximum injection time: 22 ms; normalized AGC target: 100%). Peptides were fragmented using a normalized HCD collision energy of 30%.

Bioinformatics and statistical analysis

Mass spectrometry data files were analyzed using Spectronaut (Biognosys) by direct DIA analysis (dDIA). MS/MS spectra were searched against the Uniprot proteome reference from Homo Sapiens database UP000005640 using standard settings. Enzyme was set to trypsin in a specific mode. Carbamidomethyl (C) was set as a fixed modification, and oxidation (M), acetyl (protein N-term), deamidation (N), and Gln-> pyro-Glu as variable modifications. Identifications were filtered by a 1% Q-value.

The obtained quantitative data were exported to Perseus software (version 1.6.15.0) (Tyanova S, et al. Nat Methods. 2016) for statistical analysis and data visualization. For quantitative analysis, unpaired Student’s t test was used for direct comparisons. Statistical significance was set at p-value lower than 0.05 in all cases and 1% peptide FDR threshold was considered. Differentially expressed proteins were considered significant when their absolute fold change was below 0.77 (downregulated proteins) and above 1.3 (up-regulated proteins) in linear scale.

Minicircles

Minicircles consisting almost only of the gene of interest (GOI) derived from parental plasmids (PP) with that GOI, e.g. an antibiotic resistance marker and an origin of replication within E.coli (ori), as well as two special signal sequences right and left of the GOI. An intra-molecular recombination process (Darquet et al., 1997; Bigger et al., 2001: Chen et al., 2003; Jechlinger et al., 2004) separated the parental plasmid (PP) into a miniplasmid (MP) and a minicircle (MC) (Scheleef, 2013). The GOI (plus one recombination signal sequence element) is present within the MC, that is circular and finally results in only the GOI and the remaining sequence element deriving from the recombination event in a supercoiled circular molecule.

The production of MC DNA is carried out in 2 major steps: the cultivation in a bioreactor and the purification by specific chromatographic steps. The cultivations were carried out at 37°C in a MBR bioreactor (MBR BIO REACTOR, Switzerland) with 5 L, pH adjusted to 7.0 with 2 M sodium hydroxide solution and 2 M phosphoric acid. The air flow rate was fixed at 5 L/min. The oxygen concentration of 60% was controlled by varying the stirrer speed. LB-medium was used without addition of any antibiotics. The bioreactor was inoculated with 50 mL of an E. coli K12 culture

transformed with the parental plasmid PP and grown in LB-medium for approximately 15 h. The recombinase expression was induced at an OD600 » 4 by adding L-arabinose. After 1 h of further growth, cells were harvested by centrifugation, frozen and purified by the PlasmidFactory contract manufacturing service (Bielefeld, Germany). After initial optimization runs in a 5 L scale further production runs were carried out in larger scales e.g. 20 L or above. In these cases, the pre-culture was scaled-up in a linear way. After successful recombination, the MC was separated from the MP. This was done by a series of chromatography steps, including an affinity chromatography step separating MP and MC. The approach selectively binds a sequence motif (identification sequence) with the purpose of separating this from a mixture of different DNAs (Gossen et al., 1993). The recombination product (MC and MP) was further purified by affinity chromatography as previously described (Mayrhofer et al., 2008). The sequence specific DNA binding was optimized with different ionic strength and pH values and resulted in a highly purified supercoiled monomeric MC product.

Biodistribution study

RVG-EVs were incubated at room temperature for 30 minutes with 5 µM DiR (D12731, Invitrogen). EVs were pellet by ultracentrifugation at 120000g 60 minutes. A washing step was then preformed by re-suspending the pellet in 25 ml PBS and subsequent ultracentrifugation at 120000g 60 minutes. RVG-Evs were resuspended in 5% glucose and injected into the tail vein. 24 hours after injection mice were sacrificed and tissues were fisher in 4% paraformaldehyde for 48 hours, after fixation organs were conserve in PBS. Ex vivo fluorescence imaging was performed using the IVIS Spectrum system (PerkinElmer Inc., MA, USA) on day 2 post-organ harvest. Fixed samples were imaged with excitation at 745 nm and emission at 800 nm for the DiR reporter. To maximize signal sensitivity, images were acquired using auto-illumination settings, including auto-exposure time, pixel binning of 8, f/Stop of 2, and a field of view (FoV) of 13.2 cm.

SUPPLEMENTAL REFERENCES

- Bigger BW, Tolmachov O, Collomber JM, Fragkos M, Palaszewski I, Coutelle C. An araC-controlled bacterial cre expression system to produce DNA minicircle vectors for nuclear and mitochondrial gene therapy. J Biol Chem. 2001; 276: 23018-27.

- Chen S. Ultrafast one-pass FASTQ data preprocessing, quality control, and deduplication using fastp. iMeta 2023; 2: e107. https://doi.org/10.1002/imt2.107.

- Chen ZY, He CY, Ehrhardt A, Kay MA. Minicircle DNA vectors devoid of bacterial DNA result in persistent and high-level transgene expression in vivo. Mol Ther. 2003; 8: 495-500.

- Gossen JA, de Leeuw WJF, Molijn AC, Vijg J. Plasmid rescue from transgenic mouse DNA using lacI repressor protein conjugated to magnetic beads. Biotechniques 1993; 14:624–629.

- Jechlinger W, Azimpour Tabrizi T, Lubitz W, Mayrhofer P. Minicircle DNA immobilized in bacterial ghosts: in vivo production of safe non-viral DNA delivery vehicles. J Mol Microbiol Biotechnol 2004; 8: 222-31.

- Mayrhofer P, Blaesen M, Schleef M, Jechlinger W. Minicircle-DNA production by site specific recombination and protein-DNA interaction chromatography. J Gene Med. 2008; 10: 1253–1269.

- Okonechnikov K, Conesa A, García-Alcalde F. Qualimap 2: advanced multi-sample quality control for high-throughput sequencing data. Bioinformatics, 2015.

- Schleef M. (ed) (2013) Minicircle and Miniplasmid DNA vectors – the future of non-viral and viral gene transfer. Wiley-Blackwell, Weinheim.

SUPPLEMENTARY FIGURES:

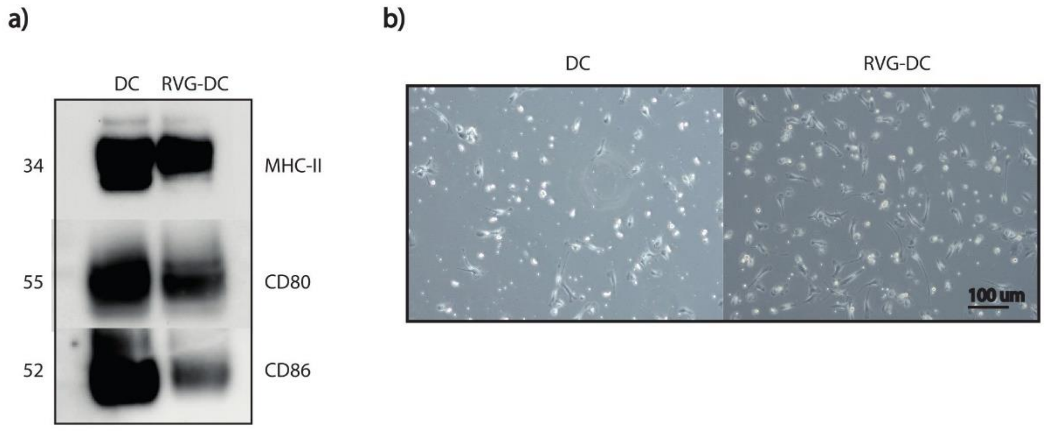

Supplementary Fig. 1. Human dendritic cells characterization. (a) Western blot detection of dendritic cell markers MHC-II, CD80 and CD86 in control dendritic cells and RVG dendritic cells. (b) Characteristic images of dendritic cells and RVG dendritic cells in culture.

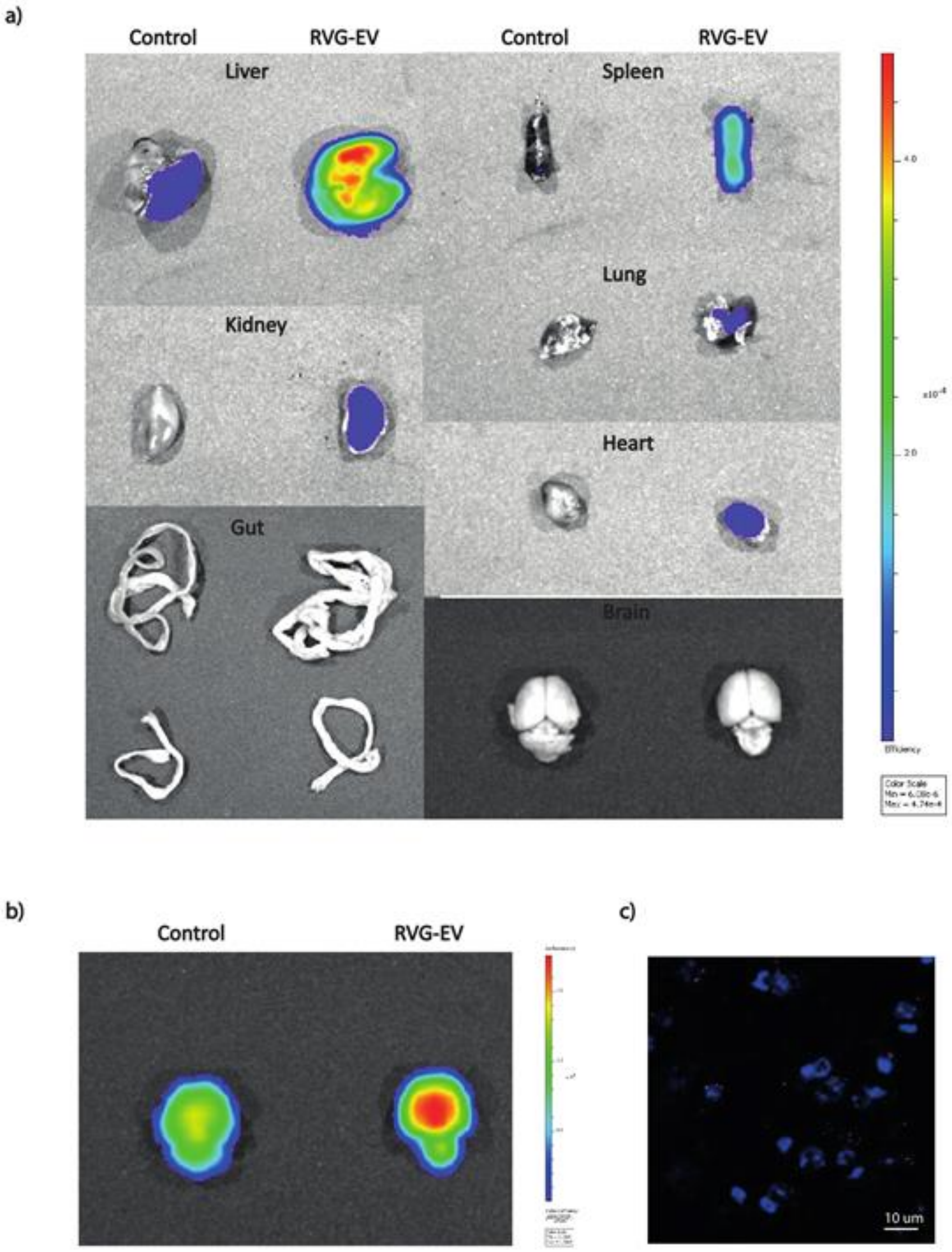

Supplementary Fig. 2. In vivo organ biodistribution of human RVG-EVs. (a) Ex vivo representative images of major organs absolute fluorescent signal using the same scale. (b) Representative image of mouse brains, harvested 24 hour post-injection of vehicle or RVG-EVs. (c) Flourescent signal of DiR labelled RVG-Evs in brain harvested 24 hour post-injection of RVG-EVs.

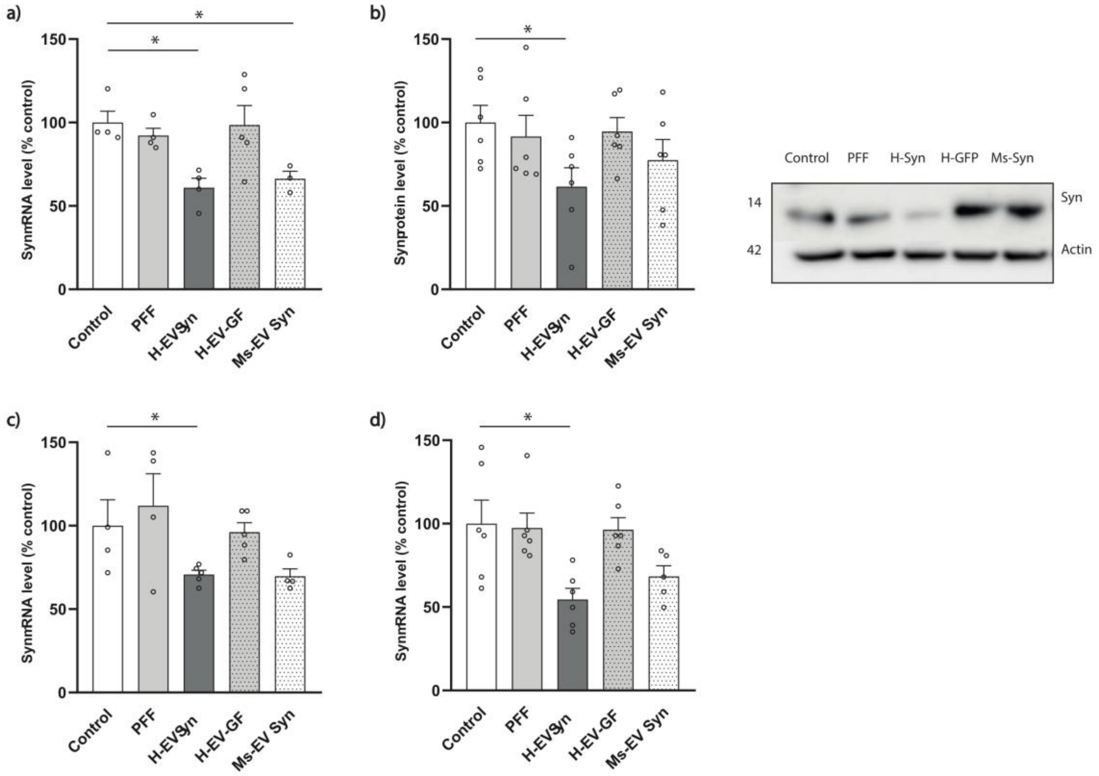

Supplementary Fig. 3. Alpha-synuclein downregulation in contralateral brain regions of mice treated with anti-alpha-synuclein shRNA-MC delivered by h-RVG-EV. Analyses of alpha-synuclein mRNA expression (a) and protein (b) levels normalized to actin in contralateral midbrain. Analyses of alpha-synuclein mRNA expression levels normalized to actin in contralateral striatum (c) and cortex (d). Typical western blot is shown. Data are expressed as mean ± SEM (n = 6). *p < 0.05, one-way ANOVA, statistical analyses compared to control mice.

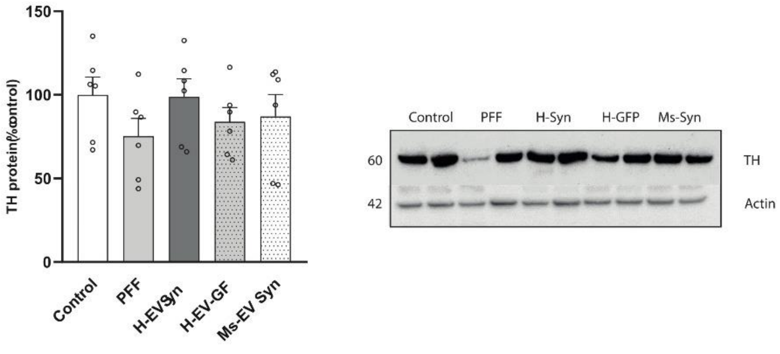

Supplementary Fig 4. Quantification of TH protein levels in midbrain. Quantification of TH protein levels normalized to beta-actin in ipsilateral midbrain. Data are expressed as mean ± SEM (n = 6).

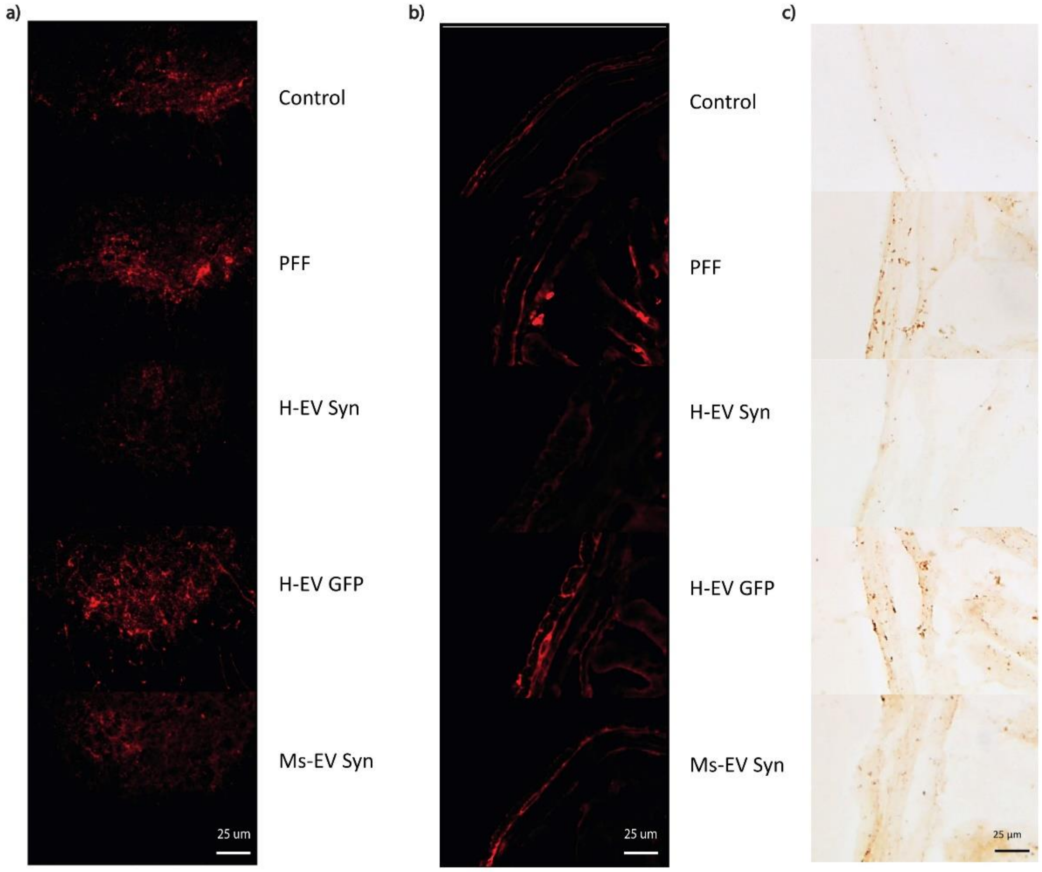

Supplementary Fig 5. Immunofluorescence images of total alpha-synuclein in cord (a) and intestinal (b) sections and immunohistochemical images of phosphorylated alpha-synuclein in intestine (c).

SUPPLEMENTARY TABLES:

Supplementary Table 1. Changes in gene expression. Transcriptomic analysis of contralateral cortex from control (CTRL), alpha-synuclein PFFs treated mice (PFF) following anti-alpha-synuclein shRNA-MC RVG-EV treatment (SYN) or anti GFP shRNA-MC RVG-EV treatment (GFP).

TEST VS REF	# DOWN	# UP	# TOTAL
GFP vs CTRL	3	1	4
PFF vs CTRL	1	0	1
SYN vs CTRL	3	0	3
PFF vs GFP	0	1	1
SYN vs GFP	0	0	0
SYN vs PFF	0	0	0

Supplementary Table 2. Inflammatory cytokine levels in PFF alpha-synuclein treated mice analyzed 90 days after the first treatment. Blood samples were collected at the end of the study and were analysed for four different cytokines. Levels of TNFα, IL-6, IFNɣ and IL-1β (pg/mL) were measured by ELISA. Values are mean +/- SEM, ND non detectable. There were no significant changes in mean level of any cytokine analysed.

	CONTROL	PFF	H-EV Syn	H-EV GF	Ms-EV Syn
TNFα (pg/mL)	ND	ND	ND	ND	ND
IL-6 (pg/mL)	29,66	30,02	33,9	26,23	25,23
INFɣ (pg/mL)	ND	ND	ND	3,8	2,6
IL-1β (pg/mL)	18,9	14,5	25,7	15,22	24,12

UNCROPED WESTERN BLOTS

Figure 1C

LAMP-2b

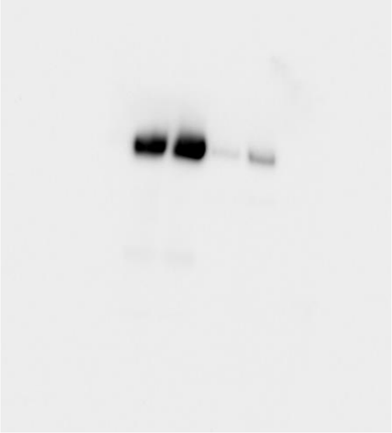

hsc-70

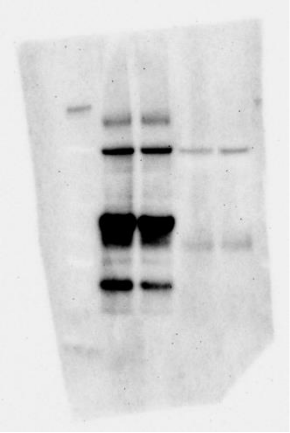

Flotillin-1

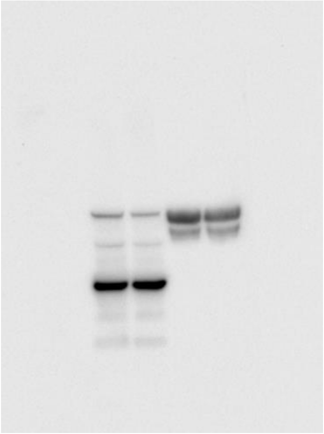

CD9

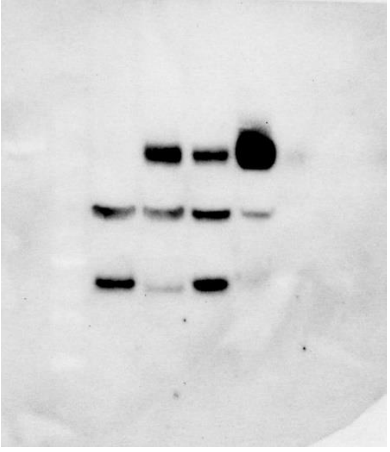

CD63

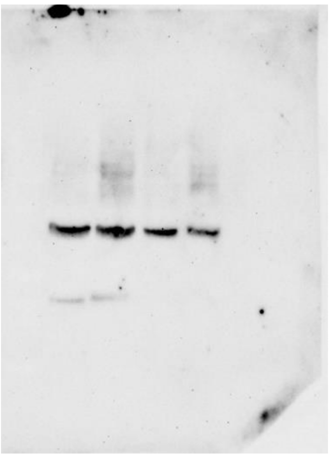

Figure 1F

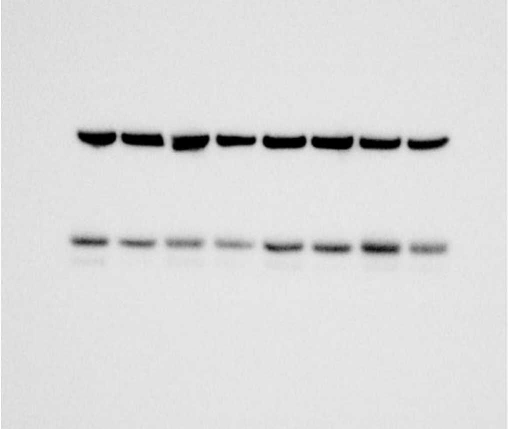

Actin

Synuclein

Figure 2B

Alpha-synuclein

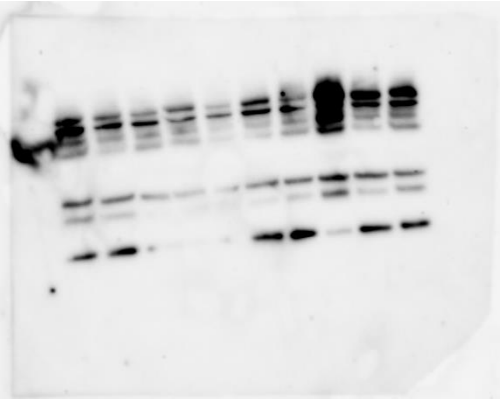

Actin

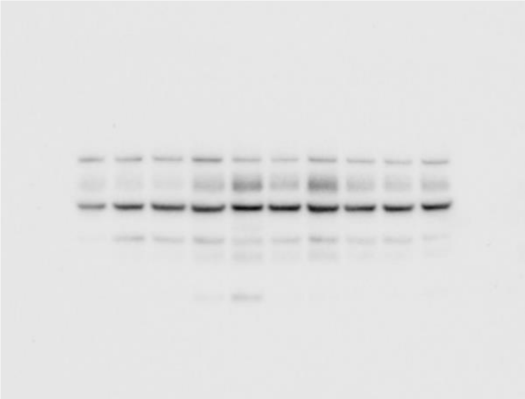

Figure 2D

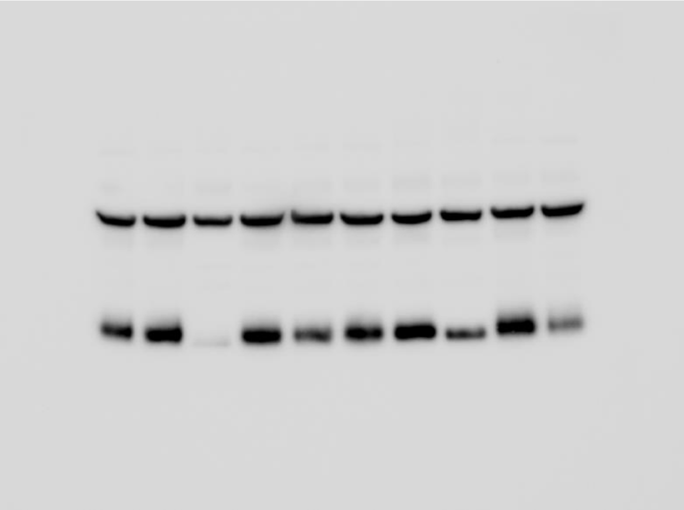

Actin

Synuclein

Figure 2F

Alpha-synuclein

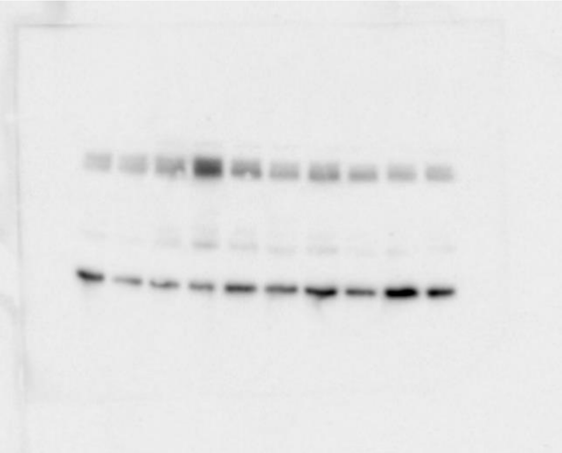

Actin

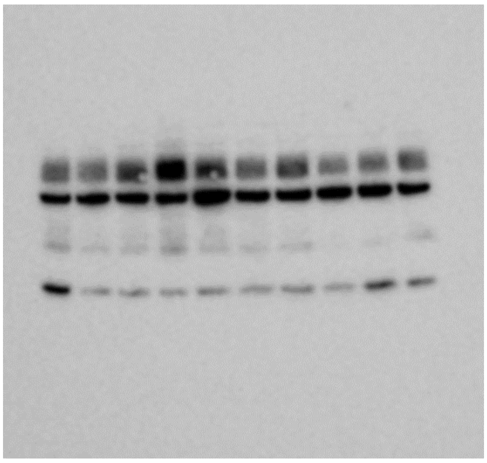

Figure 6B

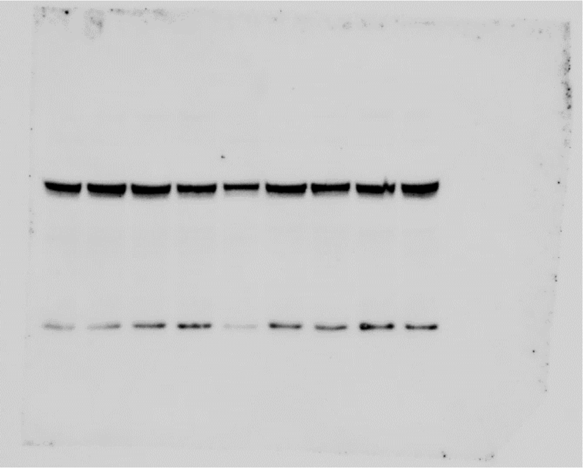

Actin

Synuclein

Figure 6D

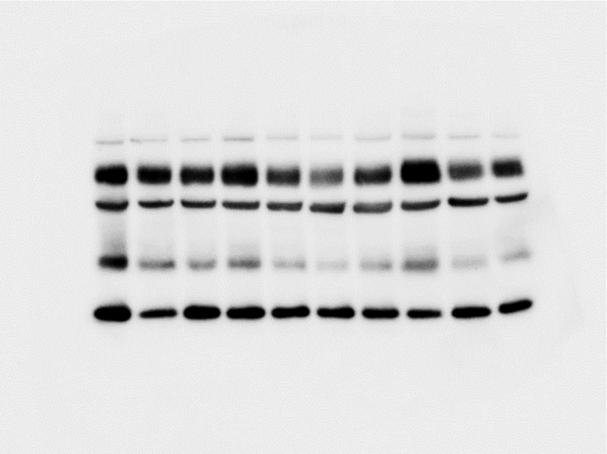

Actin

Synuclein

Figure 6F

Alpha-synuclein

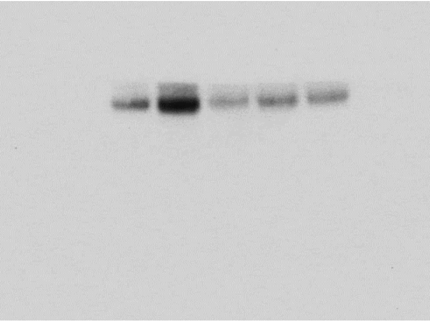

Actin

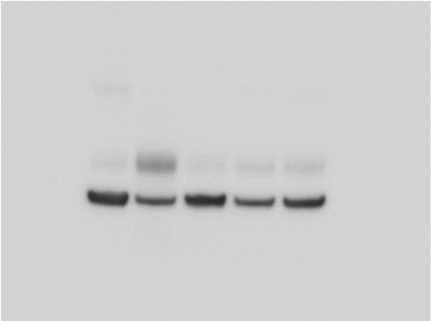

Supplementary Fig 1

MHC-II

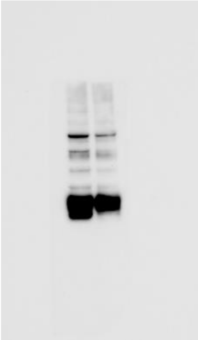

CD80

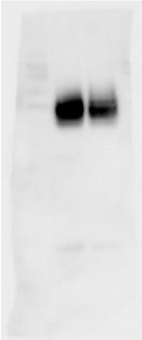

CD86

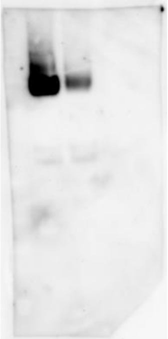

Supplementary Fig 3

Alpha-synuclein

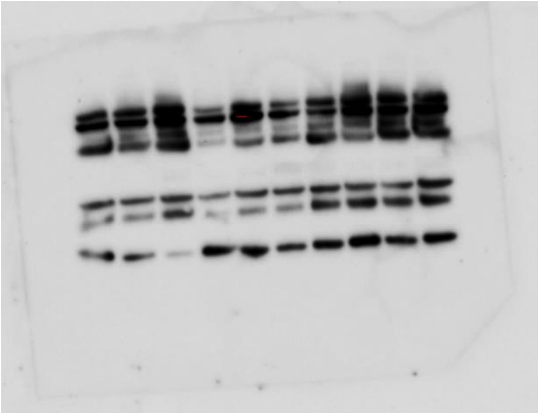

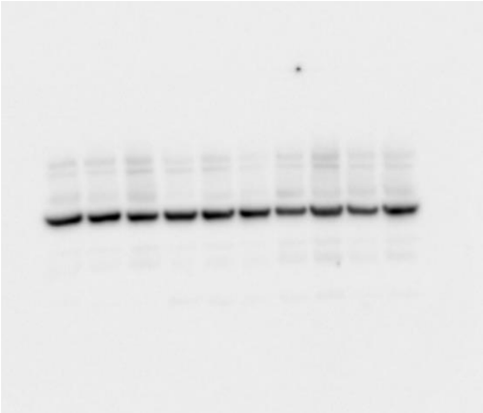

Supplementary Fig 4

TH

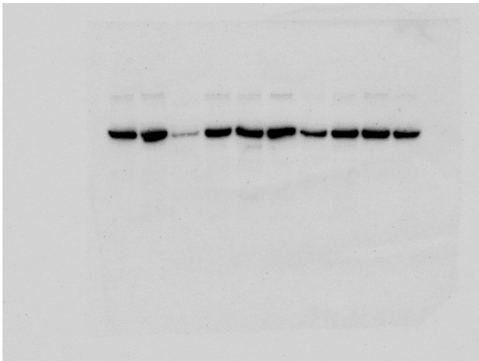

Actin

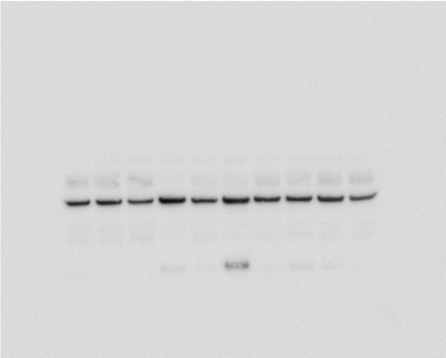

Supplement: Supplementary file 1 — Additional file 1. Supplementary Methods. Fig. S1. Human dendritic cells characterization. Fig. S2. In vivo organ biodistribution of human RVG-EVs. Fig. S3. Alpha-synuclein downregulation in contralateral brain regions of mice treated with anti-alpha-synuclein shRNA-MC delivered by h-RVG-EV. Fig. S4. Quantification of TH protein levels in midbrain. Fig. S5. Immunofluorescence images of total alpha-synuclein in cord and intestinal sections and immunohistochemical images of phosphorylated alpha-synuclein in intestine. Table S1. Changes in gene expression. Table S2. Inflammatory cytokine levels in PFFs alpha-synuclein treated mice analyzed 90 days after the first treatment. Uncropped Western blots. [file 40035_2025_484_MOESM1_ESM.pdf]
